# Attenuation of Wnt signaling by miR-27a-5p–GFPT2–HBP axis via metabolic reprogramming in colorectal cancer

**DOI:** 10.1186/s13062-026-00746-y

**Published:** 2026-03-03

**Authors:** Jiantao Jiang, Hongxin He, Xiaopeng Wang, Shangkun Jin, Wenmin Xiao, Yun Xie, Enhao Wei, Chenxin Qian, Jing Fu, Jianmin Wang, Chunkang Yang, Ruirong Lin

**Affiliations:** 1 Clinical Oncology School of Fujian Medical University, Fujian Cancer Hospital, Fuzhou, Fujian 350014 P.R. China; 2 College of Chemistry, Fuzhou University, Fuzhou, Fujian 350108 P.R. China; 3 Innovation Center for Cancer Research, Clinical Oncology School of Fujian Medical University, Fujian Cancer Hospital, Fuzhou, Fujian 350014 P.R. China; 4 Department of Thoracic Oncology Surgery, Clinical Oncology School of Fujian Medical University, Fujian Cancer Hospital, Fuzhou, Fujian 350014 P.R. China; 5 Fujian Provincial Key Laboratory of Tumor Biotherapy, Clinical Oncology School of Fujian Medical University, Fujian Cancer Hospital, Fuzhou, Fujian 350014 P.R. China

**Keywords:** Colorectal cancer, miR-27a-5p, GFPT2, Hexosamine biosynthetic pathway, O-GlcNAcylation, β-catenin, Wnt signaling.

## Abstract

**Background:**

Wnt signaling is a key driver of colorectal cancer (CRC) progression, yet directly inhibiting it remains a major challenge. MicroRNAs (miRNAs) are small noncoding RNAs that post-transcriptionally regulate gene expression, thereby modulating oncogenic pathways. However, the role of miR-27a-5p and its underlying mechanisms in CRC remains largely unknown.

**Methods:**

Bioinformatics analyses and paired clinical CRC specimens were used to evaluate miR-27a-5p expression levels and their association with prognosis. CCK-8, colony formation, wound healing, Transwell invasion, and epithelial–mesenchymal transition (EMT) marker analysis were performed to assess the effects of miR-27a-5p on the malignancy of CRC cells. The potential underlying mechanisms were investigated using dual-luciferase reporter assays, RNA-seq, HPLC-UV, immunoprecipitation/co-immunoprecipitation and immunofluorescence. Xenograft models were used to evaluate the in vivo role of miR-27a-5p in CRC.

**Results:**

miR-27a-5p was downregulated in CRC, and its low expression correlated with poorer prognosis. miR-27a-5p directly targeted GFPT2, the rate-limiting enzyme of the hexosamine biosynthetic pathway (HBP), thereby decreasing intracellular uridine 5′-diphosphate N-acetyl-D-glucosamine (UDP-GlcNAc) levels and global protein O-linked β-N-acetylglucosaminylation (O-GlcNAcylation), which in turn reduced β-catenin O-GlcNAcylation, inhibited its nuclear accumulation, and suppressed its transcriptional activity, leading to attenuation of Wnt signaling. Restoring miR-27a-5p expression in CRC cells suppressed proliferation, migration, invasion, and EMT, whereas GFPT2 overexpression or glucosamine supplementation partially reversed the inhibited malignant behaviors. Conversely, β-catenin knockdown attenuated the malignant phenotypes and expression of EMT/Wnt targets induced by miR-27a-5p inhibition, supporting a β-catenin-dependent mechanism. In mouse xenografts, treatment with the O-GlcNAc transferase (OGT) inhibitor OSMI-1 attenuated the accelerated tumor growth driven by miR-27a-5p inhibition, supporting an O-GlcNAcylation-dependent mechanism in vivo.

**Conclusion:**

These findings reveal a novel miR-27a-5p–GFPT2–HBP axis that links metabolic reprogramming to Wnt signaling in CRC by suppressing β-catenin activity through the reduction of UDP-GlcNAc-dependent O-GlcNAcylation, thereby restraining CRC progression. This suggests that targeting this axis could attenuate Wnt signaling and slow CRC progression.

**Graphical Abstract:**

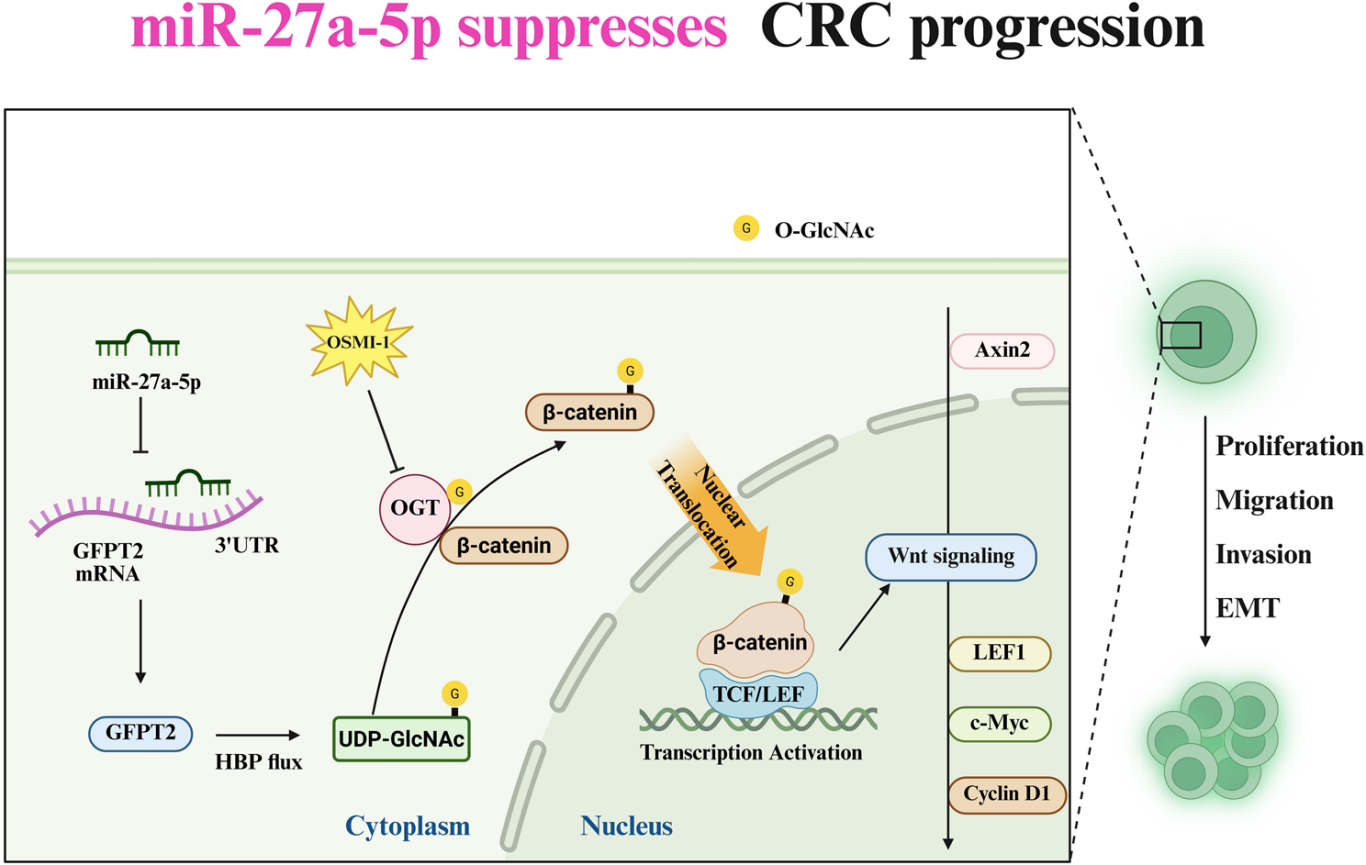

**Supplementary Information:**

The online version contains supplementary material available at 10.1186/s13062-026-00746-y.

## Introduction

Colorectal cancer (CRC) is the third most commonly diagnosed malignancy and the second leading cause of cancer-related deaths worldwide. In 2022, over 1.9 million new cases and 900 000 deaths were reported [1]. Despite advances in screening and treatment, CRC is often detected at advanced stages, when therapeutic options are limited [2]. Metastatic disease, in which tumor spread is the primary cause of CRC-related mortality, remains particularly lethal [3]. A hallmark of CRC—observed in over 90% of cases—is aberrant activation of the Wnt signaling pathway [4]. However, clinically targeting this canonical oncogenic pathway remains challenging [5], necessitating the search for alternative regulatory nodes that modulate Wnt signaling in tumor cells. MicroRNAs (miRNAs), ~ 22-nucleotide noncoding RNAs that post-transcriptionally modulate gene expression, are recognized as key regulators of carcinogenesis [6]. They target specific mRNAs and function as oncogenes (oncomiRs) or tumor suppressors depending on the context [7]. Through seed-mediated targeting and interactions with transcriptional, epigenetic, and metabolic regulators, miRNAs govern cell-fate programs such as proliferation, apoptosis, epithelial–mesenchymal transition (EMT), stemness, immune evasion, and therapy response [8]. For example, miR-21 acts as a prototypical oncomiR, repressing tumor-suppressor genes such as PDCD4 and PTEN to promote growth and invasiveness [9], whereas miR-34a, a p53-induced tumor suppressor, enforces cell-cycle arrest and apoptosis [10]. miR-27a-5p has emerged as a tumor suppressor across multiple malignancies, including gastric cancer [11], Wilms tumor [12], prostate cancer [13], and non–small cell lung cancer (NSCLC) [14]. In our institutional paired CRC cohort, miR-27a-5p expression was significantly higher in adjacent non-tumorous mucosa than in tumors; however, its role and underlying mechanisms in CRC progression remain unclear.

Cancer cells undergo metabolic reprogramming to redirect nutrients to biosynthetic pathways that support signaling processes. The hexosamine biosynthetic pathway (HBP) is a branch of glucose–glutamine metabolism that generates uridine 5′-diphosphate N-acetyl-D-glucosamine (UDP-GlcNAc), which serves as the donor substrate for protein O-linked β-N-acetylglucosaminylation (O-GlcNAcylation) [15]. Glutamine: fructose-6-phosphate amidotransferase (GFAT; encoded by GFPT1/2) catalyzes the first committed, rate-limiting step of the HBP, converting fructose-6-phosphate and L-glutamine to glucosamine-6-phosphate [16]. Subsequently, O-GlcNAc transferase (OGT) catalyzes the transfer of O-GlcNAc from the HBP end-product UDP-GlcNAc to the serine/threonine residues of substrate proteins [17]. O-GlcNAcylation regulates protein activity through various mechanisms, including the modulation of protein–protein interactions, protein stability, and subcellular localization [18], thereby affecting cell signaling, transcription, and energy utilization [19, 20]. Critically, β-catenin, the central effector of Wnt signaling [21], is regulated by O-GlcNAc modification. O-GlcNAcylation of β-catenin at specific residues, such as threonine-41, competes with phosphorylation, preventing proteasomal degradation, stabilizing β-catenin [22], and enhancing its transcriptional activity [23].

Thus, we propose a miR-27a-5p–GFPT2–HBP axis in which miR-27a-5p suppresses GFPT2-dependent HBP flux and β-catenin O-GlcNAcylation, thereby limiting Wnt signaling in CRC. Therapeutic modulation of this axis may offer a novel strategy to attenuate Wnt-driven colorectal tumor growth.

## Results

### miR-27a-5p is downregulated in CRC, and its low expression is associated with poor prognosis

**Fig. 1:**
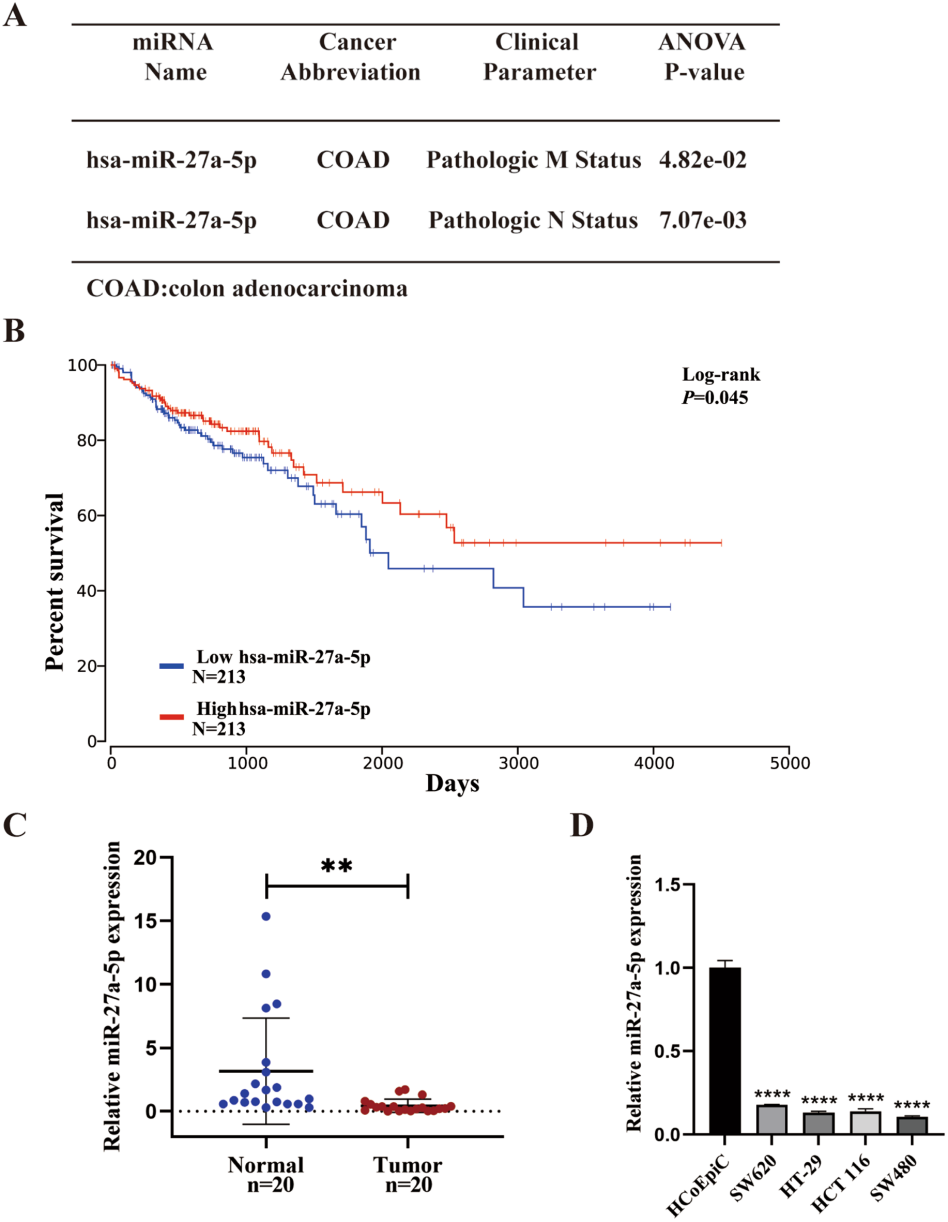
Low miR-27a-5p levels in CRC are associated with poor clinical outcome. (**A**) Relationship between tumor miR-27a-5p expression and clinicopathologic stage in COAD (OncomiR; TCGA-COAD). (**B**) Kaplan–Meier (log-rank) overall survival (OS) curves for COAD patients stratified by tumor miR-27a-5p (high vs. low; median cutoff; n_high = 213, n_low = 213; OncoLnc; TCGA-COAD). (**C**) qRT-PCR analysis of miR-27a-5p expression in paired tumors vs. adjacent non-tumorous mucosa from CRC patients. (**D**) qRT-PCR analysis of miR-27a-5p expression in CRC cell lines and normal colonic epithelial cells (HCoEpiC). **P < 0.01, ****P < 0.0001

As miRNAs are recognized regulators of CRC biology and outcomes, we examined the clinical and prognostic significance and expression pattern of miR-27a-5p. In the TCGA-COAD (The Cancer Genome Atlas–Colon Adenocarcinoma) dataset, miR-27a-5p expression varied across pathologic M and N categories (Fig. 1A). Kaplan–Meier analysis showed longer overall survival (OS) in the high miR-27a-5p expression group (Fig. 1B). In our cohort, paired qRT-PCR confirmed lower miR-27a-5p expression in tumors than in adjacent non-tumorous mucosa (Fig. 1C). miR-27a-5p levels were also reduced in CRC cell lines compared with normal colonic epithelial cells (HCoEpiC) (Fig. 1D). These findings indicate that miR-27a-5p is downregulated in CRC, and its low expression correlated with a poorer prognosis.

### miR-27a-5p suppresses proliferation, migration, invasion, and EMT in CRC cells

**Fig. 2:**
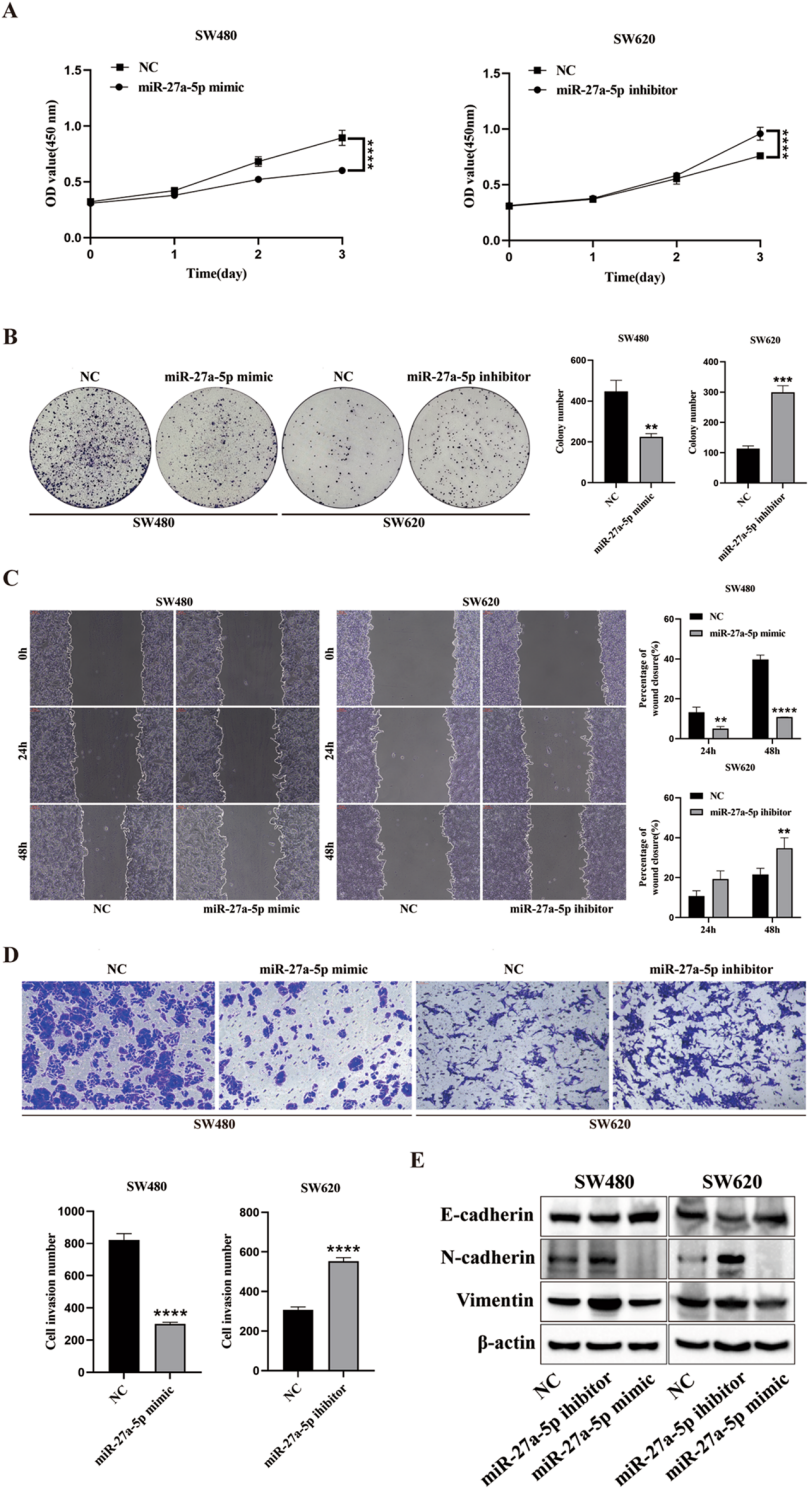
miR-27a-5p restrains proliferation, migration, invasion, and EMT in CRC cells. SW480 cells were transfected with the miR-27a-5p mimic, and SW620 cells were transfected with the miR-27a-5p inhibitor; NC served as the control. (**A**–**B**) CCK-8 and colony formation assays were performed to assess cell proliferation. (**C**) Wound-healing assays were used to assess cell migration. (**D**) Transwell assays were used to assess cell invasion. (**E**) Western blotting was used to measure EMT markers (E-cadherin, N-cadherin, and Vimentin). **P < 0.01, ***P < 0.001, ****P < 0.0001

We used SW480 (low basal miR-27a-5p) and SW620 (high basal miR-27a-5p) cells to define the functional role of miR-27a-5p. SW480 cells were transfected with a miR-27a-5p mimic, and SW620 cells were transfected with a miR-27a-5p inhibitor. Cell Counting Kit-8 (CCK-8) assays demonstrated that the miR-27a-5p mimic suppressed proliferation in SW480 cells, whereas the miR-27a-5p inhibitor enhanced proliferation in SW620 cells (Fig. 2A). Colony formation assays revealed fewer colonies with the mimic and more colonies with the inhibitor (Fig. 2B). Wound-healing assays demonstrated slower wound closure with the miR-27a-5p mimic in SW480 cells, whereas faster closure occurred in SW620 cells (Fig. 2C). Transwell invasion assays further revealed that the mimic decreased, whereas the inhibitor increased, invading cells (Fig. 2D). At the molecular level, Western blotting demonstrated that the miR-27a-5p mimic upregulated E-cadherin and downregulated N-cadherin and Vimentin, while the inhibitor produced the opposite pattern in both cell lines (Fig. 2E). Together, these data demonstrate that miR-27a-5p suppresses CRC cell proliferation, migration, invasion, and EMT.

### GFPT2 is a direct target of miR-27a-5p in CRC cells

**Fig. 3:**
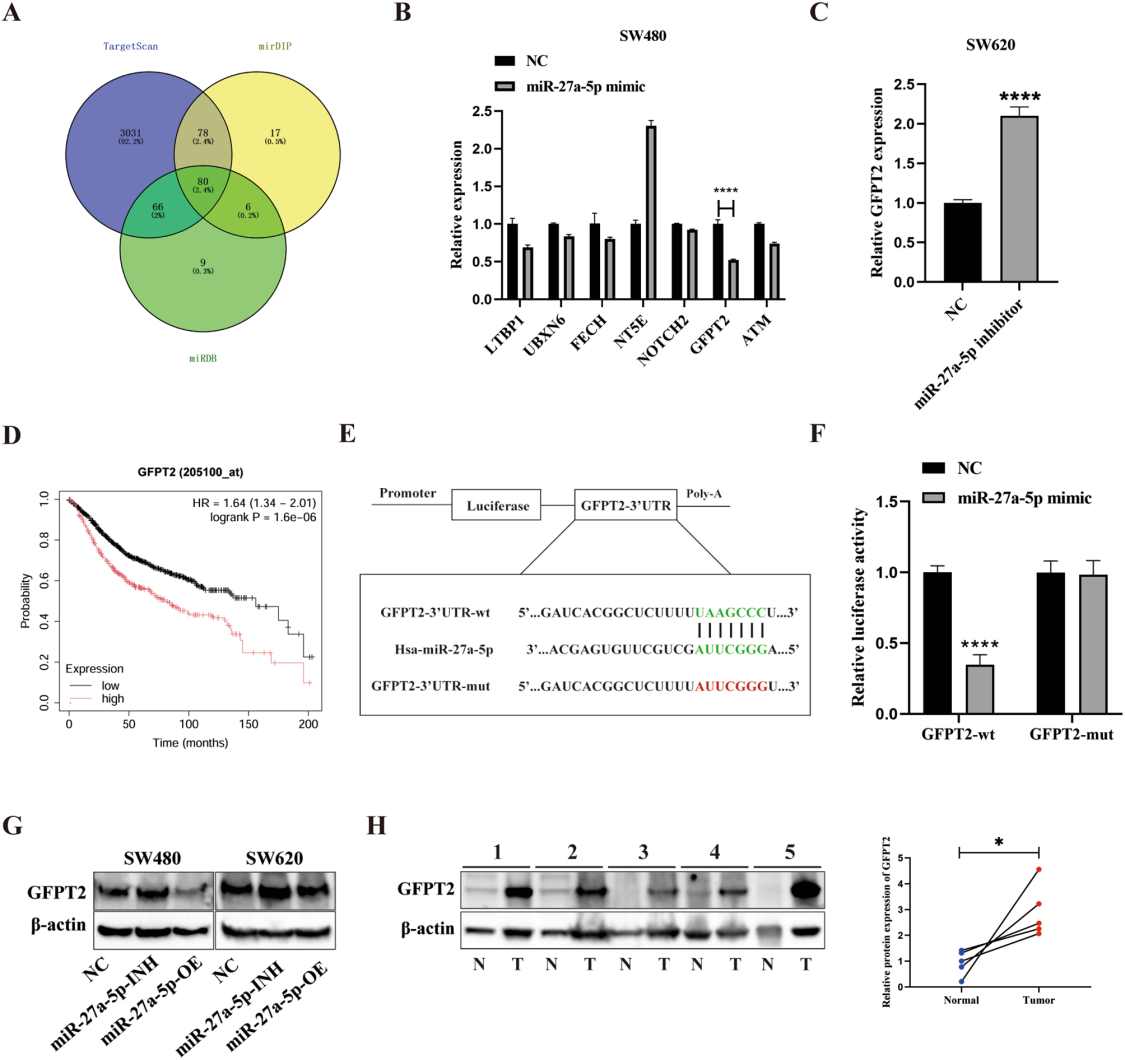
miR-27a-5p directly targets GFPT2 in CRC cells. (**A**) Venn diagram of predicted miR-27a-5p targets from TargetScan, miRDB, and mirDIP; functional filtering of the overlap to nominate candidates for validation. (**B**–**C**) Representative qRT-PCR screening of candidate targets in SW480 cells after transfection with the miR-27a-5p mimic or NC, and qRT-PCR assessment of GFPT2 levels in SW620 cells after transfection with the inhibitor or NC. (**D**) Kaplan–Meier (log-rank) analysis of OS stratified by GFPT2 expression in CRC. (**E**) Schematic of GFPT2 3′UTR luciferase reporters carrying wt or seed-site mut sequences. (**F**) Dual-luciferase assay in cells co-transfected with NC or the miR-27a-5p mimic and GFPT2-wt or GFPT2-mut reporters. (**G**) Western blot of GFPT2 in SW480 and SW620 cells following transfection with miR-27a-5p-OE, NC, or miR-27a-5p-INH. (**H**) Western blot of GFPT2 in paired CRC tumors and adjacent non-tumorous mucosa (*n* = 5). *P < 0.05, ****P < 0.0001

We further investigated the potential mechanisms underlying the inhibitory effects of miR-27a-5p in CRC. The Venn diagram generated after intersecting the predictions from TargetScan, miRDB, and mirDIP revealed 80 genes in the shared set (Fig. 3A). Subsequent functional analysis narrowed this list to 13 putative targets. We subsequently transfected SW480 cells with either a miR-27a-5p mimic or NC. qRT-PCR screening performed across these 13 candidates revealed a consistent and significant reduction in GFPT2 expression caused by miR-27a-5p (representative results shown in Fig. 3B; full dataset in Fig. S1). Conversely, a similar experiment conducted by transfecting SW620 cells with the miR-27a-5p inhibitor significantly increased GFPT2 levels compared with NC (Fig. 3C). Kaplan–Meier analysis demonstrated that elevated GFPT2 expression was associated with shorter OS (Fig. 3D), with similar trends observed for post-progression survival and relapse-free survival (Fig. S1). To validate the direct targeting of GFPT2 by miR-27a-5p, we constructed luciferase reporters harboring the wild-type (wt) or seed-mutant (mut) GFPT2 3′ untranslated region (3′UTR) (Fig. 3E). Dual-luciferase assays showed that the miR-27a-5p mimic markedly suppressed GFPT2-wt reporter activity, whereas GFPT2-mut remained unaffected (Fig. 3F). Based on these results, stable CRC cell lines with miR-27a-5p-OE (overexpression) and miR-27a-5p-INH (inhibition) were generated. Western blotting revealed reciprocal regulation of GFPT2 by miR-27a-5p in SW480 and SW620 cells: GFPT2 protein levels were lowered by miR-27a-5p-OE and increased by miR-27a-5p-INH (Fig. 3G). Notably, the expression of GFPT1, the other GFAT isoform, remained largely unchanged at both mRNA and protein levels following miR-27a-5p modulation (Fig. S2), making GFPT1 isoform compensation unlikely and supporting the specificity of the miR-27a-5p–GFPT2 axis. In paired clinical specimens, elevated levels of GFPT2 protein were observed in tumors compared with adjacent non-tumorous mucosa (Fig. 3H). Overall, our findings identify GFPT2 as a direct and clinically relevant target of miR-27a-5p in CRC.

### GFPT2 promotes proliferation, migration, and invasion in CRC cells

**Fig. 4:**
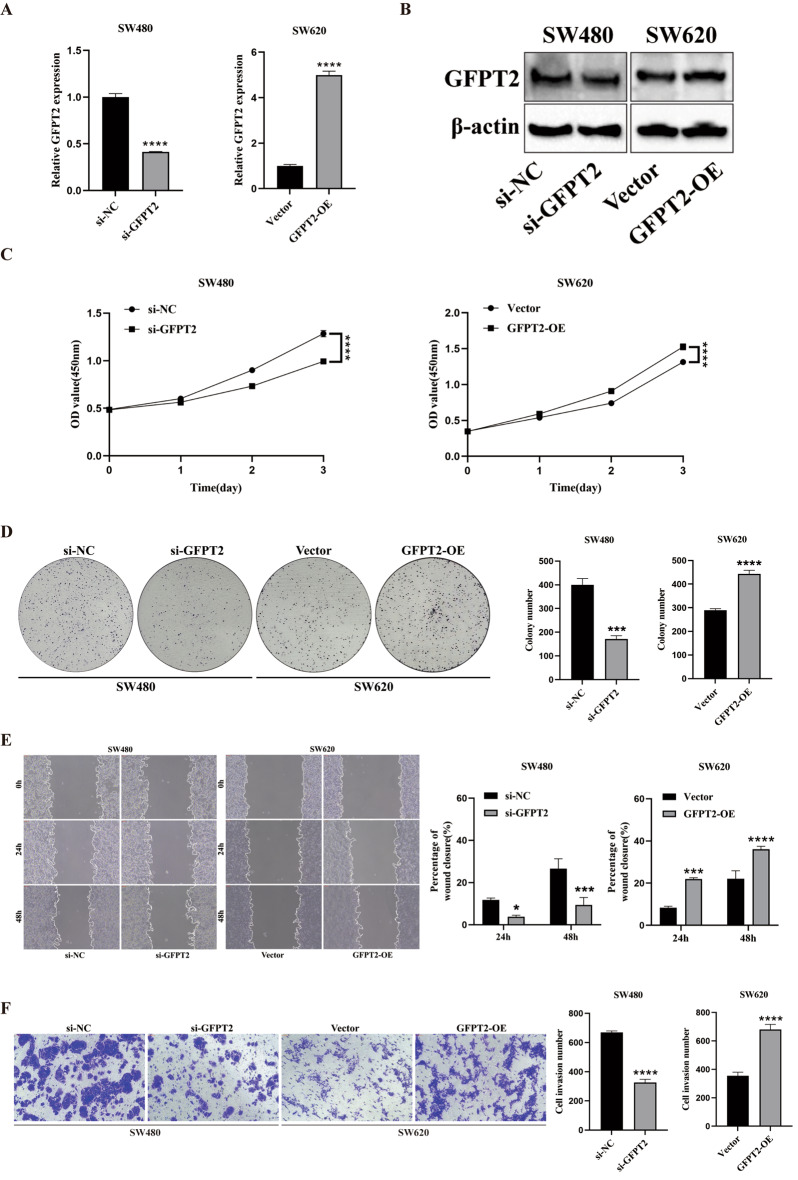
GFPT2 drives proliferation, migration, and invasion in CRC cells. SW480 cells were transfected with si-GFPT2 or si-NC, and SW620 cells were transfected with GFPT2-OE or Vector. (**A**–**B**) qRT-PCR and Western blotting confirmed efficient GFPT2 knockdown and overexpression in the indicated cells. (**C**–**D**) CCK-8 and colony formation assays were performed to assess cell proliferation. (**E**) Wound-healing assays were used to assess cell migration. (**F**) Transwell assays were used to assess cell invasion. *P < 0.05, ***P < 0.001, ****P < 0.0001

Based on prior reports linking GFPT2 to CRC aggressiveness [24] and our data indicating that miR-27a-5p targets GFPT2, we examined whether altering GFPT2 levels could recapitulate the phenotypes observed upon miR-27a-5p manipulation. For this, we transfected SW480 cells with si-GFPT2, and SW620 cells were transfected with GFPT2-OE. Efficient GFPT2 knockdown or overexpression in the indicated groups was confirmed by qRT-PCR and Western blotting (Fig. 4A and B). Functionally, CCK-8 assays showed that si-GFPT2 significantly reduced proliferation in SW480 cells, while GFPT2-OE increased it in SW620 cells (Fig. 4C). Consistently, colony formation assays revealed fewer colonies with si-GFPT2 in SW480 cells and more colonies with GFPT2-OE in SW620 cells (Fig. 4D). Wound-healing assays demonstrated that si-GFPT2 caused slower wound closure in SW480 cells, whereas GFPT2-OE accelerated it in SW620 cells (Fig. 4E). Transwell invasion assays further demonstrated a decrease in the number of invading cells with si-GFPT2 and an increase with GFPT2-OE (Fig. 4F). Taken together, these findings indicate that GFPT2 enhances proliferation, migration, and invasion in CRC cells.

### miR-27a-5p suppresses CRC progression by targeting GFPT2

**Fig. 5:**
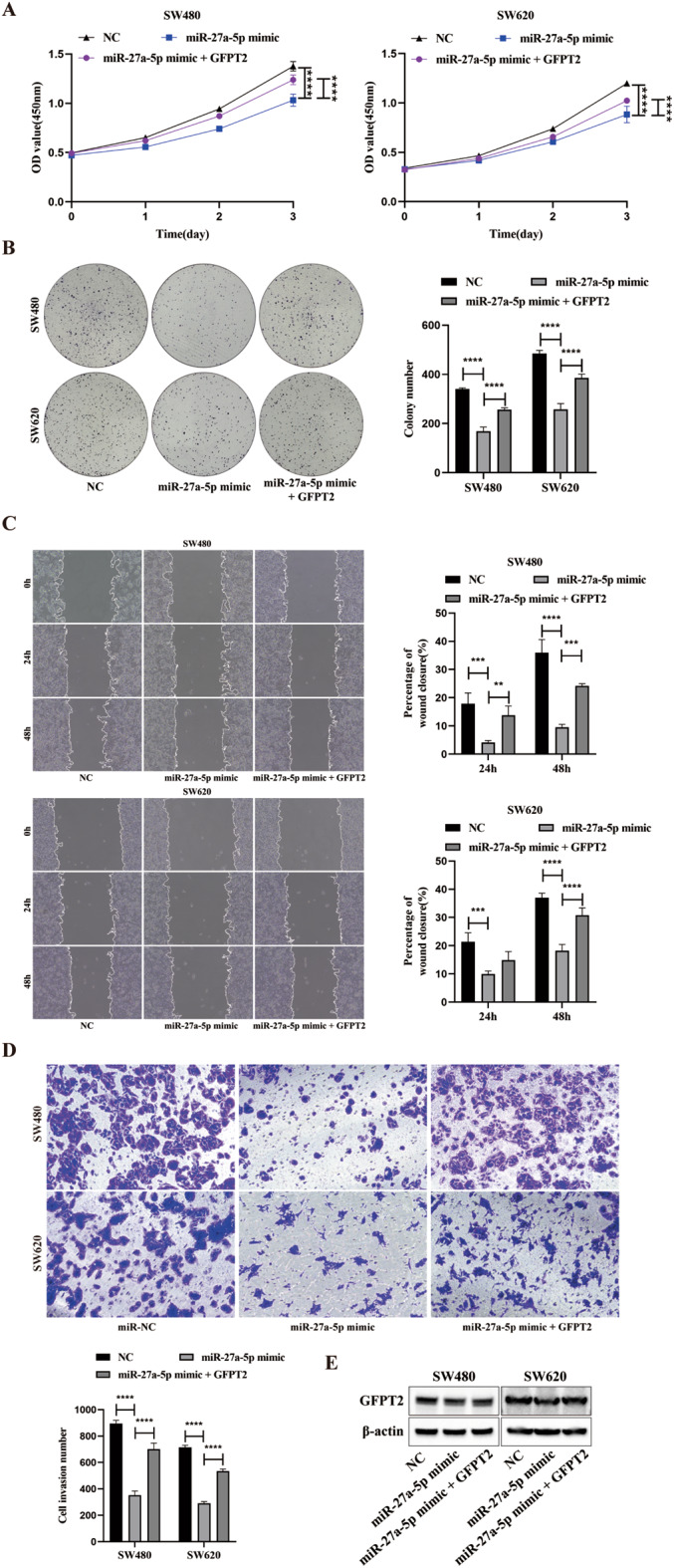
miR-27a-5p attenuates CRC progression by targeting GFPT2. SW480 and SW620 cells were transfected with the miR-27a-5p mimic alone or co-transfected with GFPT2-OE; NC served as the control. (**A**–**B**) CCK-8 and colony formation assays were performed to assess cell proliferation. (**C**) Wound-healing assays were used to assess cell migration. (**D**) Transwell assays were used to assess cell invasion. (**E**) Western blotting was used to confirm GFPT2 modulation under the indicated conditions. **P < 0.01, ***P < 0.001, ****P < 0.0001

To determine whether GFPT2 mediates the tumor-suppressive effects of miR-27a-5p, we performed rescue experiments in SW480 and SW620 cells. Cells were either transfected with the miR-27a-5p mimic alone or co-transfected with GFPT2-OE, with NC serving as the control. Cells transfected with the miR-27a-5p mimic exhibited reduced proliferation, slower wound closure, and decreased Transwell invasion in both cell lines, whereas GFPT2-OE partially reversed these effects (Fig. 5A–D). Western blotting confirmed efficient modulation of GFPT2 in SW480 and SW620 cells, reduced by the miR-27a-5p mimic and increased with GFPT2-OE (Fig. 5E). Thus, reduced GFPT2 expression is at least partially responsible for the tumor-suppressive effects exerted by miR-27a-5p.

### miR-27a-5p–GFPT2–HBP axis modulates β-catenin O-GlcNAcylation and Wnt signaling in CRC

**Fig. 6:**
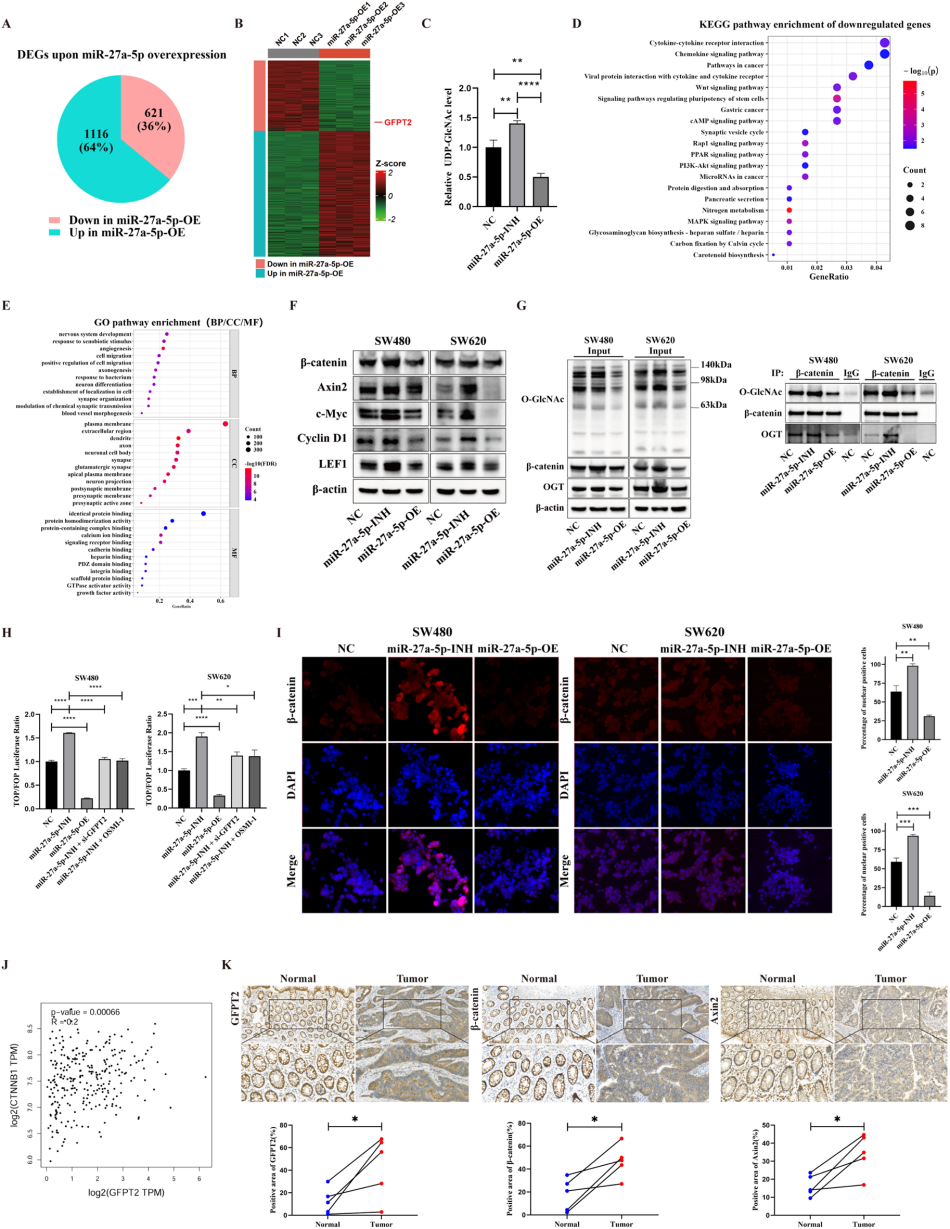
In CRC, the miR-27a-5p–GFPT2–HBP axis regulates β-catenin O-GlcNAcylation and Wnt signaling. (**A**) Summary of DEGs from RNA-seq. (**B**) Heatmap of RNA-seq profiles in SW480 cells, comparing stable miR-27a-5p-OE with NC controls across biological replicates. (**C**) HPLC–UV quantification of intracellular UDP-GlcNAc levels in SW480 cells under the indicated treatments (*n* = 3). (**D**) KEGG enrichment bubble plot for downregulated DEGs. (**E**) Gene Ontology enrichment bubble plot split by ontology (BP/CC/MF); top 12 terms per ontology. (**F**) Western blotting of SW480 and SW620 cells transfected with miR-27a-5p-INH, miR-27a-5p-OE, and NC, probed for β-catenin and its downstream targets (Axin2, c-Myc, Cyclin D1, and LEF1); β-actin, as a loading control. (**G**) β-catenin IP/co-IP in SW480 and SW620 cells. Left panels (input): global O-GlcNAc, total β-catenin, OGT, and β-actin. Right panels: β-catenin immunoprecipitates (IPs) blotted with anti–O-GlcNAc, anti–β-catenin, and anti–OGT; IgG IP, NC. (**H**) TCF/LEF-dependent transcriptional activity was measured by TOP/FOPFlash luciferase reporter assays in SW480 and SW620 cells (*n* = 3). Firefly luciferase activity was normalized to Renilla luciferase and is presented as fold change relative to the corresponding control (TOP/FOP ratio). (**I**) Immunofluorescence staining of β-catenin in SW480 and SW620 cells following miR-27a-5p modulation; representative images and quantification (percentage of nuclear positive cells, *n* = 3). Nuclei were counterstained with DAPI. Scale bars: 50 μm. (**J**) Expression correlations in the TCGA-COAD cohort between GFPT2 and CTNNB1 (β-catenin). (**K**) IHC on paired tumor and adjacent non-tumorous mucosa stained for GFPT2, β-catenin, and Axin2; representative images and quantification (percentage positive area, *n* = 5). Scale bars: 100 μm (overview), 50 μm (inset). *P < 0.05, **P < 0.01, ***P < 0.001, ****P < 0.0001

To further elucidate the underlying mechanisms, we performed RNA-seq on SW480 cells (miR-27a-5p-OE vs. NC). A total of 1 737 differentially expressed genes (DEGs) were identified, among which 621 were downregulated (36%) and 1 116 were upregulated (64%) (Fig. 6A). The heatmap demonstrates clear group separation across three biological replicates and highlights GFPT2 within the significantly downregulated cohort (Fig. 6B). Given that GFPT2 catalyzes the rate-limiting step of the HBP and thereby regulates intracellular UDP-GlcNAc levels (the donor substrate for protein O-GlcNAcylation) [15, 25], we performed HPLC–UV quantification in SW480 cells. The results demonstrated that miR-27a-5p-INH increased intracellular UDP-GlcNAc levels, whereas miR-27a-5p-OE decreased these levels (Fig. 6C; Supplementary Table S3 provides chromatographic metrics and raw readouts). To identify key genes and signaling pathways downstream of the miR-27a-5p–GFPT2–HBP axis, we performed KEGG enrichment analysis on DEGs that met our selection criteria. Genes with downregulated expression were predominantly enriched in signal transduction and cancer-related pathways, including the Wnt, PI3K–Akt, MAPK, cAMP, and Rap1 signaling pathways, as well as cytokine–cytokine receptor interactions. In addition, significant enrichment of glycosaminoglycan biosynthesis (heparan sulfate/heparin) was observed (Fig. 6D). These enrichments corroborate the established roles of O-GlcNAcylation in oncogenic signaling. Notably, Wnt signaling was consistently among the top-ranked terms across analyses, and its core components mapped to multiple enriched pathways; therefore, Wnt signaling was prioritized for subsequent analyses. Gene Ontology analysis identified three enriched modules—neuronal/synaptic (axonogenesis, synapse organization, neuron differentiation), motility/adhesion (cell migration, cadherin binding, GTPase activator activity), and vascular (angiogenesis). The overall enrichment profile aligned with Wnt biology (cadherin/β-catenin adhesion and planar cell polarity–RhoA and Ca²⁺ branches), indicating Wnt-dependent regulation of adhesion and polarity (Fig. 6E). Western blotting analysis demonstrated reciprocal modulation of Wnt signaling by miR-27a-5p: β-catenin and its downstream targets (Axin2, c-Myc, Cyclin D1, and LEF1) were upregulated by miR-27a-5p-INH and downregulated by miR-27a-5p-OE. These patterns were observed in both SW480 and SW620 cells (Fig. 6F).

Considering prior reports indicating that β-catenin undergoes O-GlcNAcylation with effects on stability and transcriptional activity [22, 23], we examined endogenous β-catenin as an O-GlcNAc-responsive signaling node downstream of the miR-27a-5p–GFPT2–HBP axis. We first assessed endogenous β-catenin O-GlcNAcylation and its association with OGT. Input lysates displayed parallel shifts in β-catenin protein and global O-GlcNAc levels, which were upregulated by miR-27a-5p-INH and downregulated by miR-27a-5p-OE, whereas the OGT expression remained unchanged (Fig. 6G, left panels). Western blotting of β-catenin immunoprecipitates (IPs) with anti–O-GlcNAc detected a specific signal absent in IgG controls. Results showed that in SW480 and SW620 cells, β-catenin–associated O-GlcNAc increased following miR-27a-5p-INH and decreased with miR-27a-5p-OE. Re-examination of the same β-catenin IPs with anti–β-catenin revealed no significant variation across conditions, confirming that changes reflected β-catenin O-GlcNAc occupancy rather than IP efficiency. Further, β-catenin IPs probed with anti–OGT detected co-precipitation of OGT, whereas IgG controls showed no signal (Fig. 6G, right panels). The amount of co-precipitated OGT varied in parallel with β-catenin–associated O-GlcNAc levels, indicating an endogenous interaction between β-catenin and OGT. To verify that β-catenin O-GlcNAcylation regulates its transcriptional activity, we assessed TCF/LEF-dependent transcriptional activity using the TOP/FOPFlash luciferase reporter assays. Consistent with the Western blotting results, the TOP/FOP luciferase ratio was significantly increased by miR-27a-5p-INH but markedly decreased by miR-27a-5p-OE in both SW480 and SW620 cells. Crucially, this hyperactivation of Wnt transcriptional output induced by miR-27a-5p inhibition was effectively abrogated by concurrent GFPT2 silencing or treatment with the OGT inhibitor OSMI-1 (Fig. 6H). Given that nuclear accumulation of β-catenin is a prerequisite for TCF/LEF-mediated transcriptional activation, we next examined the subcellular localization of β-catenin in SW480 and SW620 cells using immunofluorescence assays. The results showed that miR-27a-5p inhibition significantly promoted the nuclear accumulation of β-catenin. Conversely, miR-27a-5p overexpression markedly suppressed its nuclear accumulation (Fig. 6I). Finally, a positive correlation was observed between GFPT2 expression and CTNNB1 (β-catenin) expression in the TCGA-COAD cohort (Fig. 6J). This observation was further supported by the immunohistochemistry (IHC) of paired tumor and adjacent non-tumorous mucosa: tumors exhibited a higher percentage of positive area for GFPT2, β-catenin and the Wnt target Axin2 compared with the matched adjacent non-tumorous mucosa (Fig. 6K). Consistently, GFPT1 expression showed no significant change at either the mRNA or protein level upon OSMI-1 treatment in miR-27a-5p-INH cells (Fig. S2). These data demonstrate that the miR-27a-5p–GFPT2 axis shifts the level of β-catenin O-GlcNAcylation by modulating HBP flux and cellular O-GlcNAc levels, which in turn affects β-catenin nuclear accumulation and thereby regulates Wnt signaling in CRC.

### HBP metabolites rescue the suppressive effects of miR-27a-5p on CRC progression

**Fig. 7:**
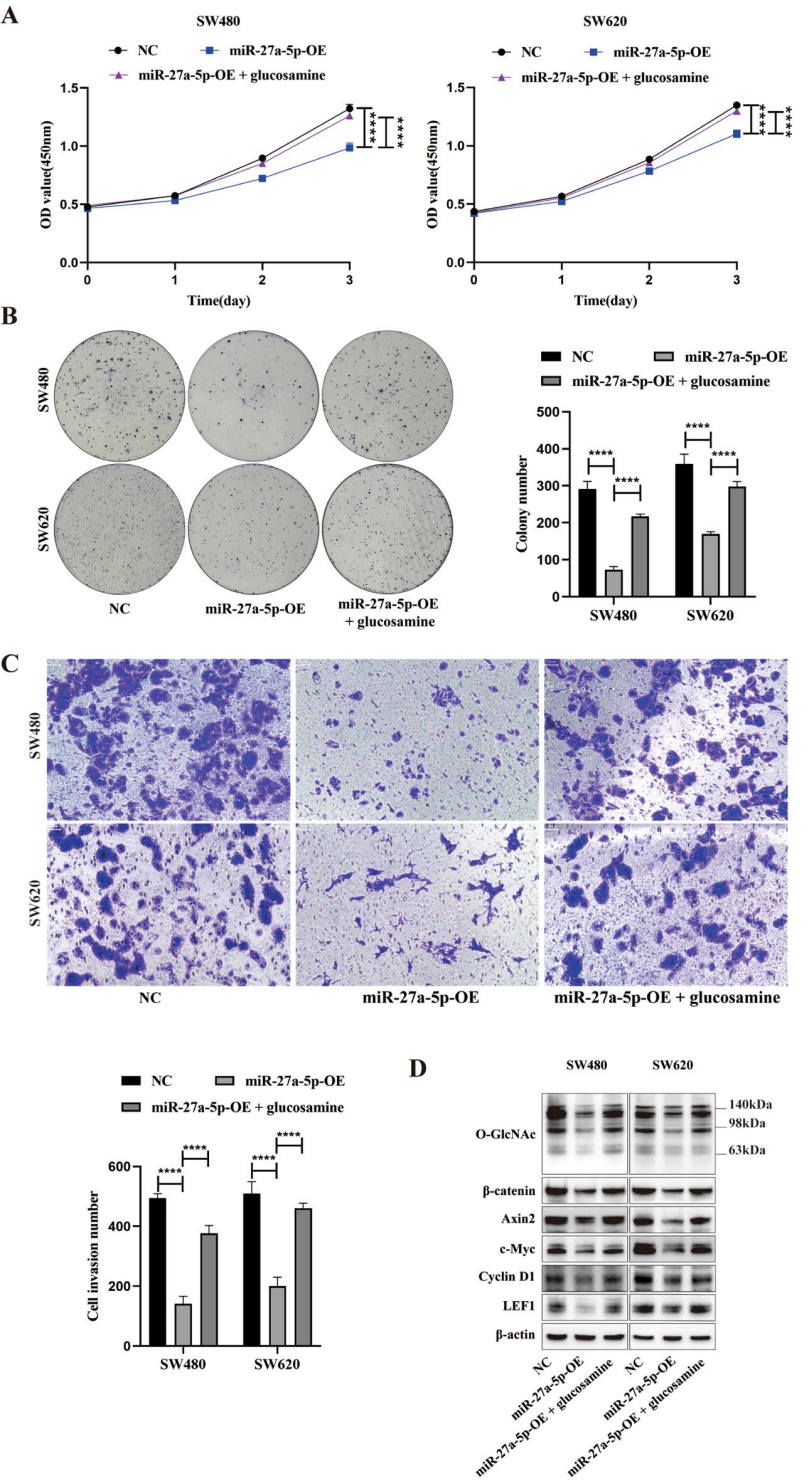
Exogenous HBP metabolites rescue the tumor-suppressive effects of miR-27a-5p on CRC progression. SW480 and SW620 miR-27a-5p-OE cells were treated with exogenous glucosamine as indicated. (**A**–**B**) CCK-8 and colony formation assays were performed to assess cell proliferation. (**C**) Transwell assays were used to assess cell invasion. (**D**) Western blotting was performed to evaluate global protein O-GlcNAcylation, β-catenin, and its downstream targets (Axin2, c-Myc, Cyclin D1, and LEF1) under the indicated conditions. ****P < 0.0001

To further validate whether the tumor-suppressive role of miR-27a-5p is mediated through the restriction of HBP flux, we performed rescue experiments by supplementing miR-27a-5p-OE cells with exogenous glucosamine—a key HBP intermediate that bypasses the rate-limiting step catalyzed by GFPT2 and replenishes cellular UDP-GlcNAc pools [26, 27]. CCK-8 and colony formation assays demonstrated that the suppressed proliferative and colony-forming capacities in miR-27a-5p-OE cells was significantly restored upon glucosamine treatment (Fig. 7A–B). Similarly, Transwell invasion assays revealed that the reduced invasive capacity induced by miR-27a-5p overexpression was markedly rescued by glucosamine supplementation (Fig. 7C). At the molecular level, Western blotting confirmed that miR-27a-5p overexpression reduced global protein O-GlcNAcylation as well as the levels of β-catenin and its downstream targets (Axin2, c-Myc, Cyclin D1, and LEF1), and that these effects were substantially reversed by glucosamine supplementation (Fig. 7D). Collectively, these data indicate that miR-27a-5p suppresses CRC progression, at least in part, by limiting HBP-derived UDP-GlcNAc levels and global protein O-GlcNAcylation, thereby attenuating Wnt signaling.

### The miR-27a-5p–GFPT2–HBP axis regulates CRC progression in a β-catenin-dependent manner

**Fig. 8:**
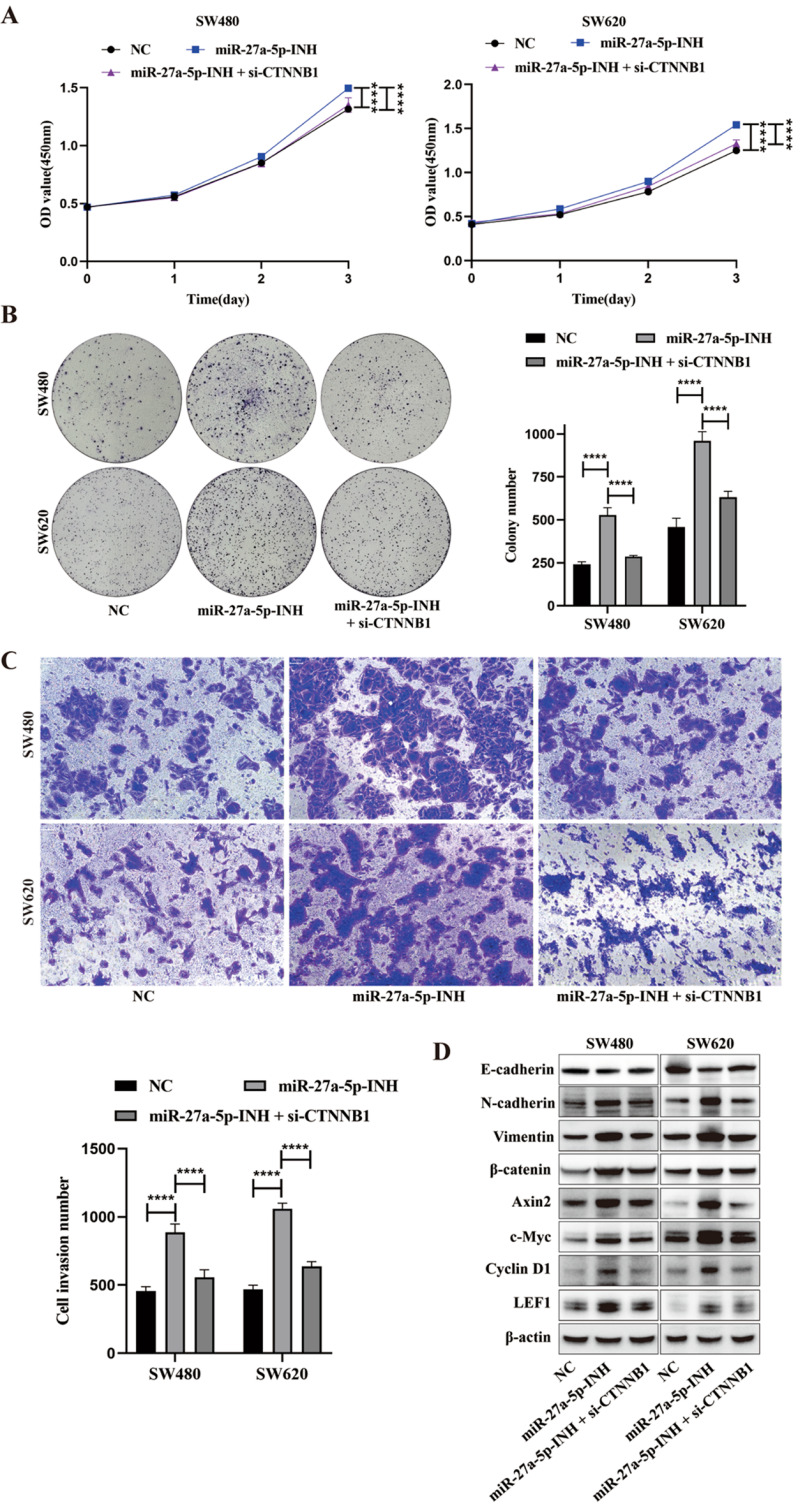
The miR-27a-5p–GFPT2–HBP axis drives CRC progression through β-catenin–dependent Wnt signaling. SW480 and SW620 miR-27a-5p-INH cells were transfected with si-CTNNB1 or si-NC as indicated. (**A**–**B**) CCK-8 and colony formation assays were performed to assess cell proliferation. (**C**) Transwell assays were used to assess cell invasion. (**D**) Western blotting was performed to evaluate EMT markers (E-cadherin, N-cadherin, and Vimentin), β-catenin and its downstream targets (Axin2, c-Myc, Cyclin D1, and LEF1) under the indicated conditions. ****P < 0.0001

To determine whether β-catenin is functionally required for the oncogenic phenotypes induced by the miR-27a-5p–GFPT2–HBP axis, we performed epistasis experiments by knocking down β-catenin with si-CTNNB1 in miR-27a-5p-INH cells. CCK-8 and colony formation assays demonstrated that the hyperproliferation observed in miR-27a-5p-INH cells was significantly attenuated upon β-catenin silencing (Fig. 8A–B). Transwell invasion assays showed that the enhanced invasive capacity in miR-27a-5p-INH cells was markedly suppressed by concurrent β-catenin knockdown (Fig. 8C). At the molecular level, Western blotting revealed that in miR-27a-5p-INH cells, the upregulation of mesenchymal markers (N-cadherin and Vimentin), β-catenin and its downstream targets (Axin2, c-Myc, Cyclin D1, and LEF1), along with the downregulation of E-cadherin, was reversed by β-catenin silencing (Fig. 8D). Collectively, these data support a model in which the miR-27a-5p–GFPT2–HBP axis promotes CRC progression and EMT, at least in part, through the β-catenin–dependent Wnt output.

### miR-27a-5p inhibition promoted tumor growth in vivo, which was attenuated by the OGT inhibitor OSMI-1

**Fig. 9:**
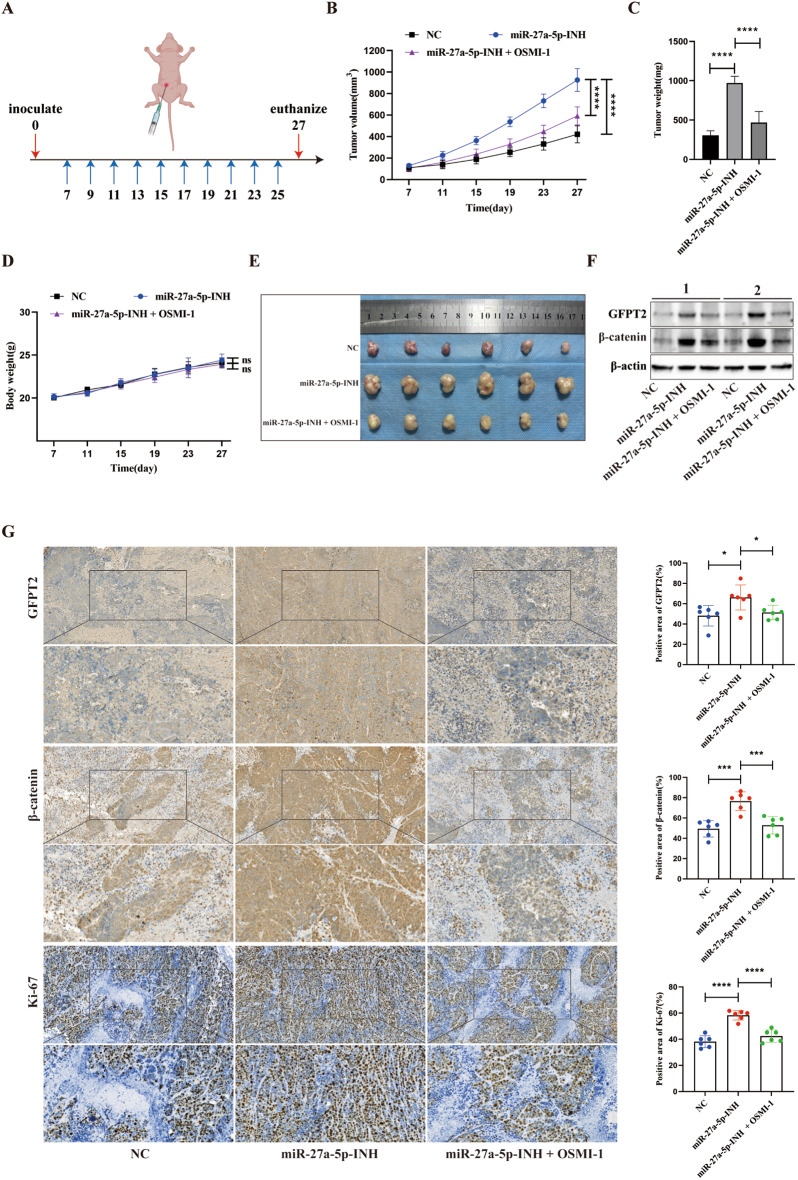
miR-27a-5p inhibition accelerates in vivo tumor growth, mitigated by the OGT inhibitor OSMI-1. SW620 cells stably expressing miR-27a-5p-INH or NC were injected subcutaneously into nude mice (*n* = 6 per group). (**A**) Beginning on day 7, mice received OSMI-1 (5 mg/kg, i.p.) or vehicle every other day for 10 doses; mice were euthanized at the endpoint. (**B**) Longitudinal tumor volume measurements. (**C**) Tumor weights at harvest. (**D**) Longitudinal monitoring of mouse body weights during the treatment period. (**E**) Photographs of tumors at harvest. (**F**) Western blotting of xenograft lysates for GFPT2 and β-catenin with β-actin as a loading control (*n* = 2). (**G**) GFPT2, β-catenin, and Ki-67 IHC on xenograft tumors; representative images and quantification (percentage positive area, *n* = 6). Scale bars: 100 μm (overview), 50 μm (inset). ****P < 0.0001

SW620 cells transfected with miR-27a-5p-INH or NC were subcutaneously inoculated into nude mice and randomly assigned to treatment groups to assess the effect of miR-27a-5p on CRC tumor growth in vivo. A subset of mice in the miR-27a-5p-INH cohort received the OGT inhibitor OSMI-1, while the remaining mice received the vehicle (Fig. 9A). Longitudinal tumor growth curves indicated that miR-27a-5p-INH accelerated tumor outgrowth compared with NC, whereas OSMI-1 significantly attenuated this effect (Fig. 9B). The tumor weights consistently confirmed these observations (Fig. 9C). To evaluate the potential systemic toxicity of OSMI-1, mouse body weights were monitored throughout the treatment period. No significant differences were observed among the groups, indicating that the dosing regimen was well tolerated and that the reduced tumor growth was unlikely to be attributable to compromised animal health (Fig. 9D). Photographs of tumors at harvest are shown (Fig. 9E). Western blotting of xenograft lysates demonstrated that miR-27a-5p-INH increased GFPT2 and β-catenin levels compared with NC, whereas OSMI-1 partially restored both toward control levels (Fig. 9F). To validate these findings in situ, we performed IHC analysis of xenograft tumors, which revealed that miR-27a-5p inhibition increased GFPT2 and β-catenin immunoreactivity, and these increases were attenuated by OSMI-1 treatment. Consistently, Ki-67 immunoreactivity was elevated in the miR-27a-5p-INH group and was reduced upon OSMI-1 treatment (Fig. 9G). Collectively, these data confirm that miR-27a-5p restrains CRC tumor growth in vivo and that the growth advantage conferred by miR-27a-5p inhibition is mitigated by the pharmacologic inhibition of O-GlcNAcylation with OSMI-1.

## Discussion

In this study, we identified miR-27a-5p as a tumor-suppressive miRNA that is downregulated in CRC. Restoration of miR-27a-5p expression in CRC cells markedly inhibited proliferation, migration, invasion, and EMT. Mechanistically, our data demonstrate that by directly targeting GFPT2, which catalyzes the first and rate-limiting step of the HBP [25], miR-27a-5p restricts HBP flux and cellular UDP-GlcNAc levels. By repressing GFPT2, miR-27a-5p reduces intracellular UDP-GlcNAc, the sugar donor for protein O-GlcNAcylation. OGT catalyzes the transfer of O-GlcNAc from UDP-GlcNAc to serine/threonine residues on target proteins [28]. Consequently, miR-27a-5p overexpression lowers global O-GlcNAcylation, significantly reducing β-catenin O-GlcNAcylation. This post-translational change restricts β-catenin nuclear accumulation and reduces TCF/LEF-dependent transcriptional activity, thereby attenuating Wnt signaling—a pathway frequently hyperactivated in CRC [29]—as evidenced by reduced Wnt target-gene expression. Notably, the functional significance of the miR-27a-5p–GFPT2–HBP axis was substantiated through multidimensional rescue experiments. In vitro, the suppression of malignant behaviors induced by miR-27a-5p overexpression was partially rescued by GFPT2 reintroduction and by exogenous glucosamine supplementation, linking HBP flux to CRC progression. Moreover, the oncogenic phenotypes and the hyperactivation of Wnt signaling induced by miR-27a-5p inhibition were significantly attenuated by concurrent β-catenin knockdown, supporting a β-catenin–dependent mechanism downstream of this axis. The in vivo relevance of this pathway was further underscored by mouse xenograft studies, in which pharmacologic inhibition of O-GlcNAcylation via OSMI-1 significantly attenuated tumor growth accelerated by miR-27a-5p deficiency. Collectively, these findings define a miR-27a-5p–GFPT2–HBP axis that modulates β-catenin O-GlcNAcylation and Wnt signaling to regulate CRC progression. Clinically, our results align with patient data. For instance, an independent study has reported significantly lower serum miR-27a levels in CRC patients compared with healthy controls, and higher miR-27a levels were further positively correlated with advanced TNM stage, tumor recurrence, and metastasis [30]. In our study, miR-27a-5p was downregulated in tumors compared with adjacent non-tumorous mucosa. Higher miR-27a-5p levels have been associated with favorable outcomes in CRC, underscoring the potential prognostic utility of miR-27a-5p and supporting the clinical relevance of our mechanistic model.

Aberrant Wnt signaling is a central driver of colorectal tumorigenesis [29]. Most CRCs harbor APC or β-catenin–stabilizing mutations that constitutively activate Wnt signaling, driving uncontrolled proliferation and survival [31]. Within this context, miRNAs represent an upstream regulatory layer of oncogenic signaling. Prior studies have reported tumor-suppressive roles for miR-27a-5p across multiple cancers: He et al. reported that miR-27a-5p targets APEX1 to downregulate MAPK/AKT signaling and inhibit EMT and proliferation in gastric cancer [11]. Guo et al. demonstrated that miR-27a-5p targets PBOV1 in Wilms tumor, reducing proliferation, migration, and invasion while increasing apoptosis, with in vivo confirmation of reduced xenograft growth [12]. Barros-Silva et al. found that miR-27a-5p attenuates EGFR–AKT–mTOR signaling thereby suppressing oncogenic behavior [13], and Shi et al. reported its tumor-suppressive role in NSCLC via the repression of MELK and inhibition of FAK signaling [14]. Taken together, these studies support our finding that miR-27a-5p acts as a tumor suppressor in CRC.

Consistent with previous reports, GFPT2 has emerged as a driver of aggressive phenotypes in several cancers. Liu et al. identified GFPT2 upregulation in aggressive CRC, which was associated with liver metastasis and poor prognosis [24]. Szymura et al. showed that NF-κB–driven GFPT2 upregulation promotes EMT-associated migration and invasion in NSCLC [32], and Wang et al. reported frequent GFPT2 overexpression during EMT, with its silencing reducing mesenchymal markers and invasion in claudin-low breast cancer [33]. These findings collectively support the pro-tumor role of GFPT2 and align with its position within the miR-27a-5p–GFPT2–HBP pathway.

Our data demonstrate that miR-27a-5p regulates Wnt signaling through metabolic reprogramming, which adds a new mechanistic dimension to CRC biology. miR-27a-5p does not directly target Wnt components but inhibits GFPT2, thereby reducing β-catenin O-GlcNAcylation and limiting Wnt signaling. To our knowledge, this study provides the first evidence linking the miR-27 family to the HBP in cancer and the first experimental validation of direct GFPT2 regulation by a miRNA. The biological significance of this metabolic regulation is further supported by evidence from other malignancies: in pancreatic cancer, HBP hyperactivation drives aggressive behavior by maintaining β-catenin activity [34]; in serous ovarian cancer, GFPT2 upregulation increases nuclear β-catenin and induces EMT and invasion via elevated protein O-GlcNAcylation [35]. More broadly, O-GlcNAcylation has been recognized as a key mediator of oncogenic signaling: O-GlcNAcylation of β-catenin [22, 36], p65 [24, 37], and c-Myc [38, 39] promotes proliferation and invasion by enhancing their stability and activity. A recent mechanistic study in *Cell* used RNA aptamers to recruit OGT to β-catenin, showing that increased O-GlcNAcylation inhibits β-TrCP binding, stabilizes β-catenin, and enhances transcriptional activity via EZH2 recruitment [23]. These findings align with our observation that miR-27a-5p upregulation or OSMI-1 treatment reduces O-GlcNAcylation, lowering β-catenin levels and Wnt signaling, while increased HBP flux via GFPT2 overexpression yields the opposite effect. Consistent with prior studies, the β-catenin–OGT interaction was confirmed, indicating that OGT-mediated O-GlcNAcylation enhances β-catenin transcriptional activity and sustains Wnt signaling in CRC. Interestingly, we observed that pharmacologic OGT inhibition with OSMI-1 downregulated GFPT2 in vivo, consistent with studies showing reports stating that OGT blockade downregulates GFPT1 [40]. This effect may be attributable to transcriptional or post-transcriptional remodeling of the HBP rather than classic UDP-GlcNAc feedback inhibition on GFPT enzymatic activity, warranting further mechanistic investigation. Beyond these internal feedback mechanisms, the translational impact of the miR-27a-5p–GFPT2–HBP axis must be considered within the specific genetic landscape of CRC. The high prevalence of APC or CTNNB1 mutations in CRC presents a potential hurdle, as these alterations constitutively stabilize β-catenin [41]. However, our observation that Wnt target genes remain sensitive to perturbation of the miR-27a-5p–GFPT2–HBP axis in SW480 cells—despite their characteristically high basal Wnt activity driven by truncated APC [42, 43]—reveals a significant regulatory layer. This suggests that β-catenin O-GlcNAcylation provides a secondary regulatory layer that fine tunes Wnt output largely in parallel with the destruction complex. Specifically, while genetic lesions dictate the “quantity” of β-catenin by limiting its degradation, O-GlcNAcylation governs the “quality” of Wnt signaling output by modulating its nuclear occupancy and transcriptional potency, consistent with our observations of altered β-catenin nuclear accumulation and TCF/LEF-dependent transcriptional activity upon axis perturbation. Therefore, targeting the HBP/O-GlcNAcylation checkpoint may represent a viable strategy to dampen Wnt hyperactivation even in the presence of APC/CTNNB1 alterations.

Despite these strengths, our study has limitations. First, while we identified the miR-27a-5p–GFPT2–HBP axis as a central mechanism in CRC, we recognize the pleiotropic nature of miRNAs. Although GFPT2 is a major metabolic mediator supported by our rescue data, miR-27a-5p likely co-regulates additional candidate targets identified in our bioinformatic screening, including genes linked to cell-cycle control or apoptotic programs. These secondary pathways may contribute to the observed phenotypes in a context-dependent manner, indicating that the GFPT2–HBP axis is a dominant, but not exclusive, mechanism underlying miR-27a-5p–mediated tumor suppression. Second, although our assays primarily focused on β-catenin, O-GlcNAcylation also intersects with PI3K–Akt and MAPK signaling [44, 45], which could influence CRC cell behavior. Future work employing mass spectrometry–based proteomics and glycoproteomics is warranted to comprehensively map the O-GlcNAc-dependent effector landscape under miR-27a-5p regulation. While our data support our core conclusions, validation in patient-derived organoids is needed to confirm the breadth and specificity of this mechanism.

## Conclusion

In conclusion, we have delineated a miRNA-driven metabolic checkpoint wherein miR-27a-5p restrains Wnt signaling in CRC. By targeting GFPT2 to reduce HBP flux and UDP-GlcNAc levels, miR-27a-5p diminishes β-catenin O-GlcNAcylation, limits its nuclear accumulation, and reduces TCF/LEF-dependent transcriptional activity, ultimately suppressing proliferation, migration, invasion, and EMT. Given the difficulty of directly targeting Wnt signaling [46], indirect modulation via the miR-27a-5p–GFPT2–HBP axis may represent a viable approach to attenuate pathway activity and bypass some challenges and toxicities associated with Wnt inhibitors. Future research should elucidate the upstream mechanisms driving miR-27a-5p downregulation in CRC—such as epigenetic silencing, transcriptional loss, or lncRNA sponging—to inform therapeutic reactivation strategies.

## Materials and methods

### Tissue samples

Paired primary colorectal tumor tissues and adjacent non-tumorous tissues were obtained intraoperatively from 20 patients with histopathologically confirmed CRC at Fujian Cancer Hospital. None of the patients had received neoadjuvant chemotherapy or radiotherapy. Resection specimens were snap-frozen in liquid nitrogen and stored at − 80 °C until analysis. The study was approved by the Institutional Ethics Committee of Fujian Cancer Hospital and conducted in accordance with the Declaration of Helsinki; all participants provided written informed consent.

### Cell culture

Human CRC cell lines HCT116, HT-29, SW480, and SW620, together with normal human colon epithelial cells (HCoEpiC) and human embryonic kidney 293T (HEK293T) cells, were obtained from the Cell Bank of the Chinese Academy of Sciences (Shanghai, China). All cell lines were authenticated by short tandem repeat analysis, tested for mycoplasma contamination, and cultured according to the manufacturer’s protocols. 

### Cell transfection

A miR-27a-5p mimic and miR-27a-5p inhibitor with their respective NCs, si-GFPT2 and si-NC, and GFPT2-OE and other related constructs were obtained from Tsingke Biotechnology (Beijing, China). Transfections were performed using Lipofectamine 3000 (Invitrogen, Carlsbad, CA, USA) according to the manufacturer’s protocol. Cells were harvested 48 h after transfection for downstream analyses. 

### RNA extraction and qRT-PCR

Total RNA was extracted with TRIzol reagent (Invitrogen, USA) according to the manufacturer’s instructions. RNA was reverse-transcribed to cDNA. mRNA and miRNA levels were quantified by SYBR Green–based qRT-PCR according to the manufacturer’s protocols (Vazyme, Nanjing, China). Primer sequences are provided in Table S1.

### Lentiviral transduction and stable cell line generation

SW480 and SW620 cells with stably miR-27a-5p overexpression (miR-27a-5p-OE) and stably miR-27a-5p inhibition (miR-27a-5p-INH) were generated using lentiviral vectors. After lentiviral transduction, stable transfectants were selected with puromycin. Efficacy was confirmed by qRT-PCR.

### Western blot assay

Total protein was extracted from cells or tissues using RIPA buffer (Beyotime, Shanghai, China) supplemented with a protease/phosphatase inhibitor cocktail (Beyotime). Equal amounts of protein were separated on 10% SDS–PAGE and transferred to PVDF membranes. Membranes were incubated overnight at 4 °C with the corresponding primary antibodies (Supplementary Table S2). Blots were developed by chemiluminescence. 

### Cell counting kit-8 (CCK-8) assay

Cell proliferation was assessed using the CCK-8 (Meilunbio, Dalian, China). SW480 and SW620 cells were seeded into 96-well plates at 800–1000 cells/well. At the indicated time points, 10 µL CCK-8 reagent was added to each well and plates were incubated for 90 min at 37 °C; absorbance at 450 nm was measured.

### Colony formation assay

SW480 and SW620 cells were seeded in 6-well plates at 600–800 cells/well and cultured for 10–14 days. Colonies were washed with PBS, fixed with 4% paraformaldehyde, and stained with crystal violet. After air-drying, colonies (≥ 50 cells) were imaged and counted.

### Wound healing assay

SW480 and SW620 cells were seeded in 6-well plates and grown to ~ 100% confluence. A linear wound was made across the monolayer. Phase-contrast images were captured at 0, 24, and 48 h on an inverted microscope from the same marked fields. Wound area was quantified in ImageJ, and the wound-closure rate (%) = (A₀ − A_t)/A₀ × 100, where A₀ and A_t are the wound areas at 0 h and time t, respectively. 

### Transwell invasion assay

Cell culture inserts with an 8-µm pore size were pre-coated with Matrigel (MCE, Monmouth Junction, NJ, USA). SW480 and SW620 cells were resuspended in serum-free medium and seeded into the upper chamber; the lower chamber contained complete medium. After 48 h, the invaded cells were fixed with 4% paraformaldehyde, stained with crystal violet, rinsed, and counted. 

### Dual-luciferase reporter assay

A wild-type (wt) human GFPT2 3′UTR encompassing the predicted miR-27a-5p seed-matching site was synthesized (Tsingke Biotechnology), generating GFPT2-wt. A seed-mutant (mut) harboring point mutations at the seed-matching site was generated to disrupt pairing with miR-27a-5p, generating GFPT2-mut. HEK293T cells were co-transfected with wt or mut reporters together with a miR-27a-5p mimic or NC using Lipofectamine 3000. After 48 h, firefly and Renilla luciferase activities were measured. Relative luciferase activity was calculated as the firefly/Renilla ratio and normalized to the NC group. 

### Immunoprecipitation (IP) and Co-IP assay

To examine β-catenin O-GlcNAcylation and its association with OGT, IP/co-IP was performed with the Protein A/G IP (Co-IP) Kit (Biolinkedin, Shanghai, China) according to the manufacturer’s instructions. Cells were lysed on ice in the kit-supplied lysis buffer supplemented with a protease-inhibitor cocktail and Thiamet-G (an O-GlcNAcase inhibitor; MCE). Lysates were clarified; 5–10% was reserved as input and the remainder was incubated overnight at 4 °C with anti-β-catenin or species-matched control IgG. Immune complexes were captured with the kit-provided Protein A/G beads, washed, eluted, and analyzed by Western blotting. Precipitates and inputs were probed with the corresponding antibodies (Supplementary Table S2). 

### HPLC–UV quantification of UDP-GlcNAc

UDP-GlcNAc was quantified by HPLC–UV (260 nm) on a Waters 2695 system using a Thermo Scientific (Waltham, MA, USA) C18 column (250 mm × 2.5 mm) held at 30 °C. Isocratic elution was performed at 1.0 mL/min with 10 mM ammonium bicarbonate in water and acetonitrile; injection volume was 10 µL. Samples were homogenized and centrifuged. Supernatants were dried, purified, rinsed, and eluted. Quantification was performed by external calibration using peak-area response curves. Equal numbers of cells (5 × 10^7^ cells per sample) were used for the extraction of each metabolite to normalize UDP-GlcNAc measurements.

### Immunofluorescence

Cells grown on coverslips were fixed with 4% paraformaldehyde, permeabilized with 0.1% Triton X-100, and blocked with 5% BSA. Samples were incubated overnight at 4 °C with the indicated primary antibodies (Supplementary Table S2), followed by fluorophore-conjugated secondary antibodies. Nuclei were counterstained with DAPI, and images were acquired using a fluorescence microscope.

### In vivo xenograft assays

All animal procedures were approved by the Fujian Medical University Animal Ethics Committee (IACUC FJMU 2023-0074). Male BALB/c nude mice (5–6 weeks old; 15–20 g) were used. For subcutaneous xenografts, 2 × 10⁶ tumor cells in 100 µL PBS were injected into the left axilla. Tumor dimensions were measured with calipers beginning 7 days after inoculation and then every 4 days. Starting on day 7, mice received the OGT inhibitor OSMI-1 (5 mg/kg) by intraperitoneal injection (i.p.) every 2 days for 10 doses (days 7–25). On day 27, mice were euthanized; tumors were excised and weighed.

### Immunohistochemistry (IHC)

Human tissue specimens or subcutaneous xenograft tumors were formalin-fixed in 4% paraformaldehyde, embedded in paraffin, and sectioned at 4 μm. Sections were incubated overnight at 4 °C with the corresponding primary antibodies (Supplementary Table S2). Sections were counterstained with hematoxylin, dehydrated, mounted, and imaged using an upright Imager microscope.

### Chemicals and reagents

The OGT inhibitor OSMI-1 and glucosamine were purchased from MCE. For cell treatments, cells were incubated with 50 µM OSMI-1 or 2.5 mM glucosamine for 24–48 h prior to downstream analyses. An equal volume of vehicle (DMSO or PBS) was administered to the control groups.

### Statistical analysis

Statistics were performed in GraphPad Prism 9. Data are presented as mean ± SD from ≥ 3 independent experiments unless stated. Two-group comparisons used unpaired two-tailed Student’s t test (a paired t test for matched tumor versus adjacent non-tumorous mucosa). Comparisons among three or more groups were evaluated by one-way ANOVA with Tukey’s post hoc test. Correlations between continuous variables were analyzed using Spearman’s rank correlation (ρ). Significance: *P < 0.05, **P < 0.01, ***P < 0.001, ****P < 0.0001. 

## Supplementary Information

Below is the link to the electronic supplementary material.

Supplementary Material 1

Supplementary Material 2

Supplementary Material 3

## Data Availability

The data that support the findings of this study are available from the corresponding author upon reasonable request.
