# Supplementary material for: Attenuation of Wnt signaling by miR-27a-5p–GFPT2–HBP axis via metabolic reprogramming in colorectal cancer
Source: Biol Direct. 2026 Mar 3;21:44. doi: 10.1186/s13062-026-00746-y (PMC13067420; doi:10.1186/s13062-026-00746-y)
Supplement: Supplementary file 1 — Supplementary Material 1 [file 13062_2026_746_MOESM1_ESM.docx]

**Supplementary Figure:**

**Figure S1**

**
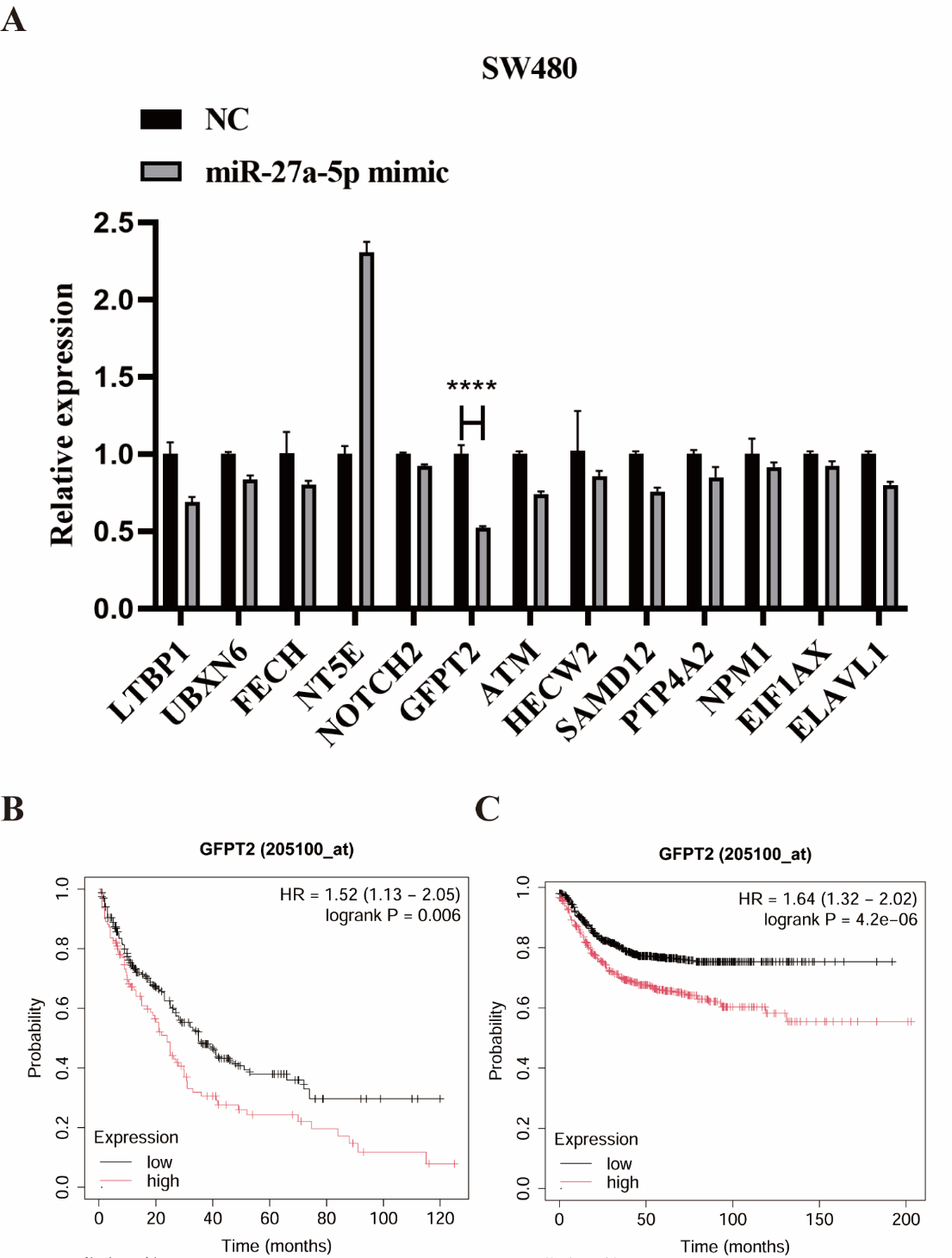
**

**Figure S1: miR-27a-5p suppresses GFPT2 among screened candidates and high GFPT2 predicts worse survival outcomes. (A)** RT–qPCR screening of 13 predicted targets in SW480 cells transfected with the miR-27a-5p mimic or NC. Relative expression was normalized to the housekeeping gene and to NC. Bars show mean ± SD (n = 3). **(B–C)** Kaplan–Meier curves for post-progression survival and relapse-free survival stratified by GFPT2 expression in two independent cohorts. **** *P* < 0.0001.

**Figure** **S2**


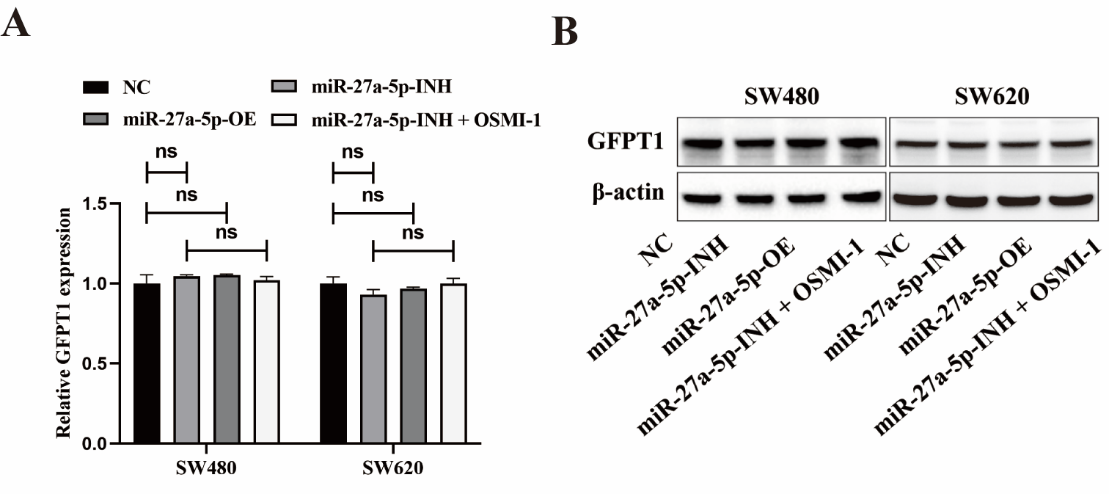


**Figure S2: GFPT1 expression is not compensatorily upregulated upon miR-27a-5p modulation or OSMI-1 treatment. (A)** qRT-PCR analysis of GFPT1 mRNA levels; data are expressed as relative fold changes. **(B)** Western blotting of GFPT1 protein levels under the indicated conditions, with β-actin serving as the loading control. ns, not significant (*P* > 0.05).
