# Supplementary material for: Attenuation of Wnt signaling by miR-27a-5p–GFPT2–HBP axis via metabolic reprogramming in colorectal cancer
Source: Biol Direct. 2026 Mar 3;21:44. doi: 10.1186/s13062-026-00746-y (PMC13067420; doi:10.1186/s13062-026-00746-y)
Supplement: Supplementary file 2 — Supplementary Material 2 [file 13062_2026_746_MOESM2_ESM.docx]

Supplementary Table:

Table S1: The primers employed in this study

| Name | Sequence |
| --- | --- |
| miR-27a-5p | F: GCGAGGGCTTAGCTGCTTG |
|  | R: AGTGCAGGGTCCGAGGTATT |
|  | SL-RT：GTCGTATCCAGTGCAGGGTCCGAGGTATTCGCACTGGATACGACTGCTCA |
| U6 | F: CTCGCTTCGGCAGCACA |
|  | R: AACGCTTCACGAATTTGCGT |
| HECW2 | F: TACCACGGCATTAGTGGAGC |
|  | R: ATCCCTTTCTTTAGCCCAACTG |
| SAMD12 | F: TGCCCATGCTGAAGGTATTAAAC |
|  | R: CGTAGCTGACTTAGCCGTCT |
| PTP4A2 | F: AGAAGGGGAGCGTTCAATTCC |
|  | R: CTCTGAAGCGTAATCGCATCTTA |
| ATM | F: TTGATCTTGTGCCTTGGCTAC |
|  | R: TATGGTGTACGTTCCCCATGT |
| FECH | F: GGAGATGTTCACGACTTCCTTC |
|  | R: GAATGGTGCCAGCTTATTCTGA |
| NT5E | F: AAGGACTGATCGAGCCACTC |
|  | R: GGAAGTGTATCCAACGATTCCCA |
| LTBP1 | F: GCTTCCGTCCAGATACATCAG |
|  | R: CTTGGTACGAGACTTGGGATTG |
| UBXN6 | F: GGAGCGCATTAACTGCCTG |
|  | R: GCTCAGCACGTAGAACTCCTC |
| NPM1 | F: GGAGGTGGTAGCAAGGTTCC |
|  | R: TTCACTGGCGCTTTTTCTTCA |
| EIF1AX | F: AACAGACGCAGGGGTAAGAAT |
|  | R: CCTGAGCATACTCCTGACCAT |
| GFPT2 | F: TTGGTCGAGAGAGTCATTCAGC |
|  | R: AAGATAGGGATCTGTTCTGTGGA |
| ELAVL1 | F: AACTACGTGACCGCGAAGG |
|  | R: CGCCCAAACCGAGAGAACA |
| NOTCH2 | F: CCTTCCACTGTGAGTGTCTGA |
|  | R: AGGTAGCATCATTCTGGCAGG |
| GFPT1 | F: AACTACCATGTTCCTCGAACGA |
|  | R: CTCCATCAAATCCCACACCAG |

Table S2: The antibodies employed in this study

| Antibody | Company | Catalog No | Notes |
| --- | --- | --- | --- |
| E-Cadherin | Abconal | A20798 | for western blotting |
| N-cadherin | proteintech | 22018-1-AP | for western blotting |
| Vimentin | proteintech | 10366-1-AP | for western blotting |
| GFPT2 | proteintech | 15189-1-AP | for western blotting and IHC |
| β-Actin | Cell Signaling | 8457 | for western blotting |
| Axin2 | Cell Signaling | 2151 | for western blotting |
| LEF1 | Cell Signaling | 2230 | for western blotting |
| Cyclin D1 | proteintech | 60186-1-Ig | for western blotting |
| c-Myc | Cell Signaling | 5605 | for western blotting |
| β-Catenin | Cell Signaling | 9582 | for western blotting |
| β-Catenin | proteintech | 51067-2-AP | for IP/ Co‐IP, IHC and IF |
| O-GlcNAc | Cell Signaling | 82332 | for western blotting |
| OGT | proteintech | 66823-1-Ig | for western blotting |
| Axin2 | Abconal | A2513 | for IHC |
| Ki-67 | AiFang Biological | AF20068 | for IHC |
| GFPT1 | Cell Signaling | 5322 | for western blotting |

Table S3: HPLC–UV quantification and chromatographic metrics of UDP-GlcNAc

| Group | Retention time | Peak area | Peak height | Peak type | Concentration |
| --- | --- | --- | --- | --- | --- |
|  | min | mAU·min | mAU |  | (µg/mL) |
| miR-27a-5p-INH–1 | 5.700 | 0.002 | 0.025 | BMB* | 0.160 |
| miR-27a-5p-INH–2 | 5.733 | 0.002 | 0.027 | BMB* | 0.171 |
| miR-27a-5p-INH–3 | 5.692 | 0.002 | 0.016 | BMB* | 0.166 |
| NC-1 | 5.642 | 0.001 | 0.020 | BMB* | 0.102 |
| NC-2 | 5.700 | 0.002 | 0.024 | BMB* | 0.130 |
| NC-3 | 5.700 | 0.001 | 0.012 | BMB* | 0.122 |
| miR-27a-5p-OE–1 | 5.742 | 0.001 | 0.013 | BMB* | 0.051 |
| miR-27a-5p-OE–2 | 5.708 | 0.001 | 0.019 | BMB* | 0.065 |
| miR-27a-5p-OE–3 | 5.658 | 0.001 | 0.014 | BMB* | 0.061 |
