# Supplementary material for: Attenuation of Wnt signaling by miR-27a-5p–GFPT2–HBP axis via metabolic reprogramming in colorectal cancer
Source: Biol Direct. 2026 Mar 3;21:44. doi: 10.1186/s13062-026-00746-y (PMC13067420; doi:10.1186/s13062-026-00746-y)

Fig. 2E

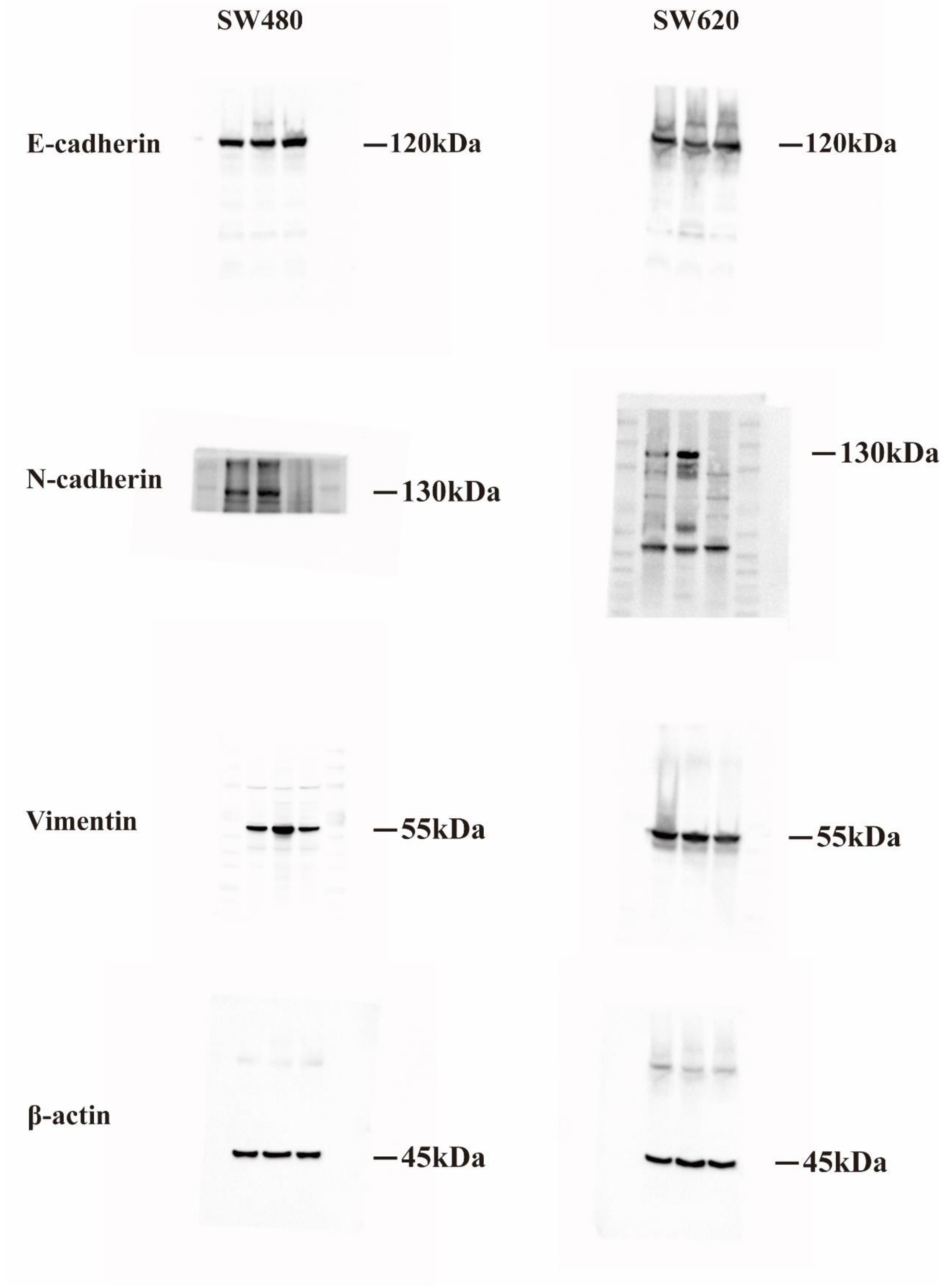
SW480
SW620
E-cadherin
—120kDa
—120kDa
N-cadherin
—130kDa
—130kDa
Vimentin
—55kDa
—55kDa
β-actin
—45kDa
—45kDa

Fig. 3G

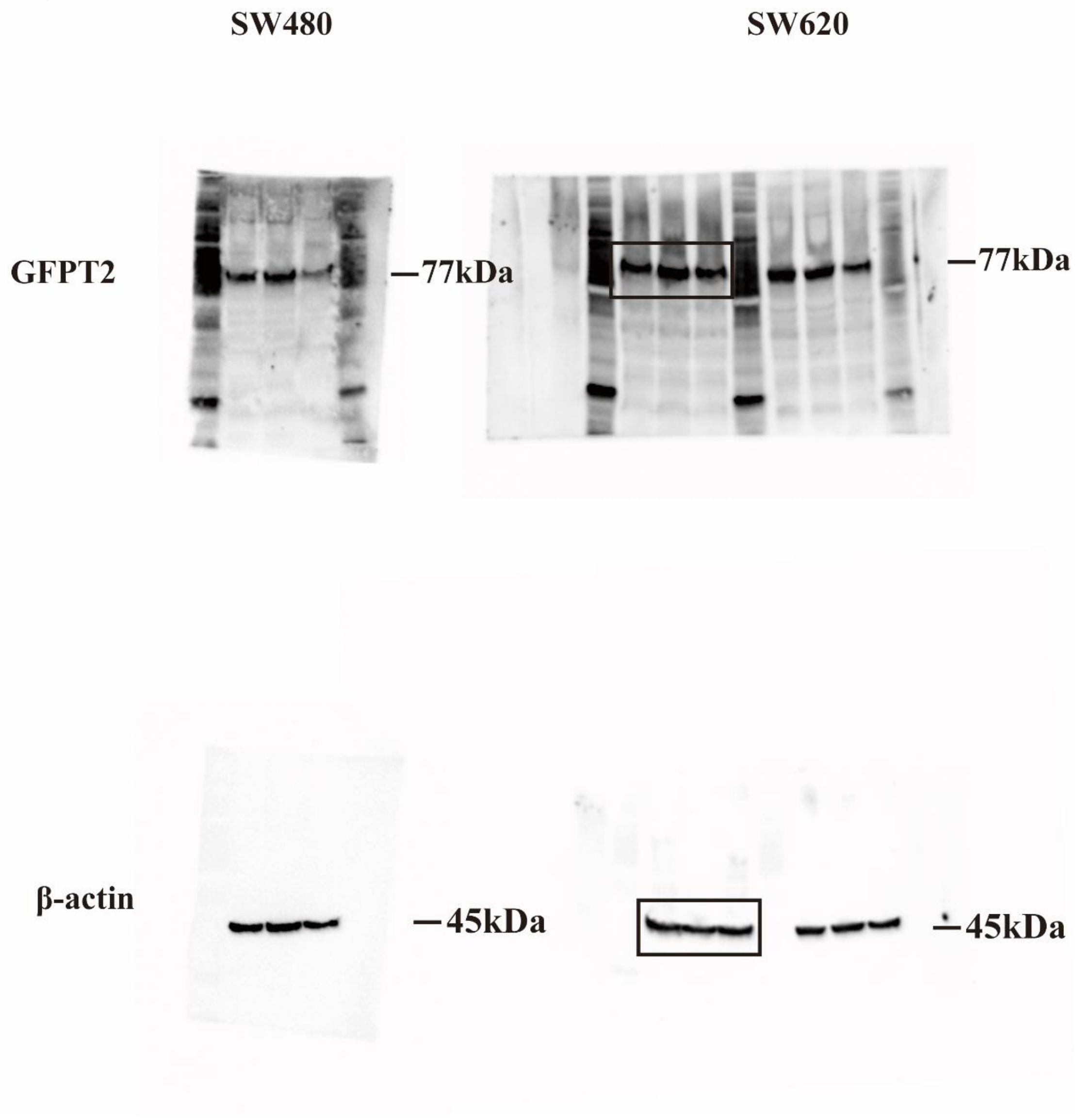

Fig. 3H

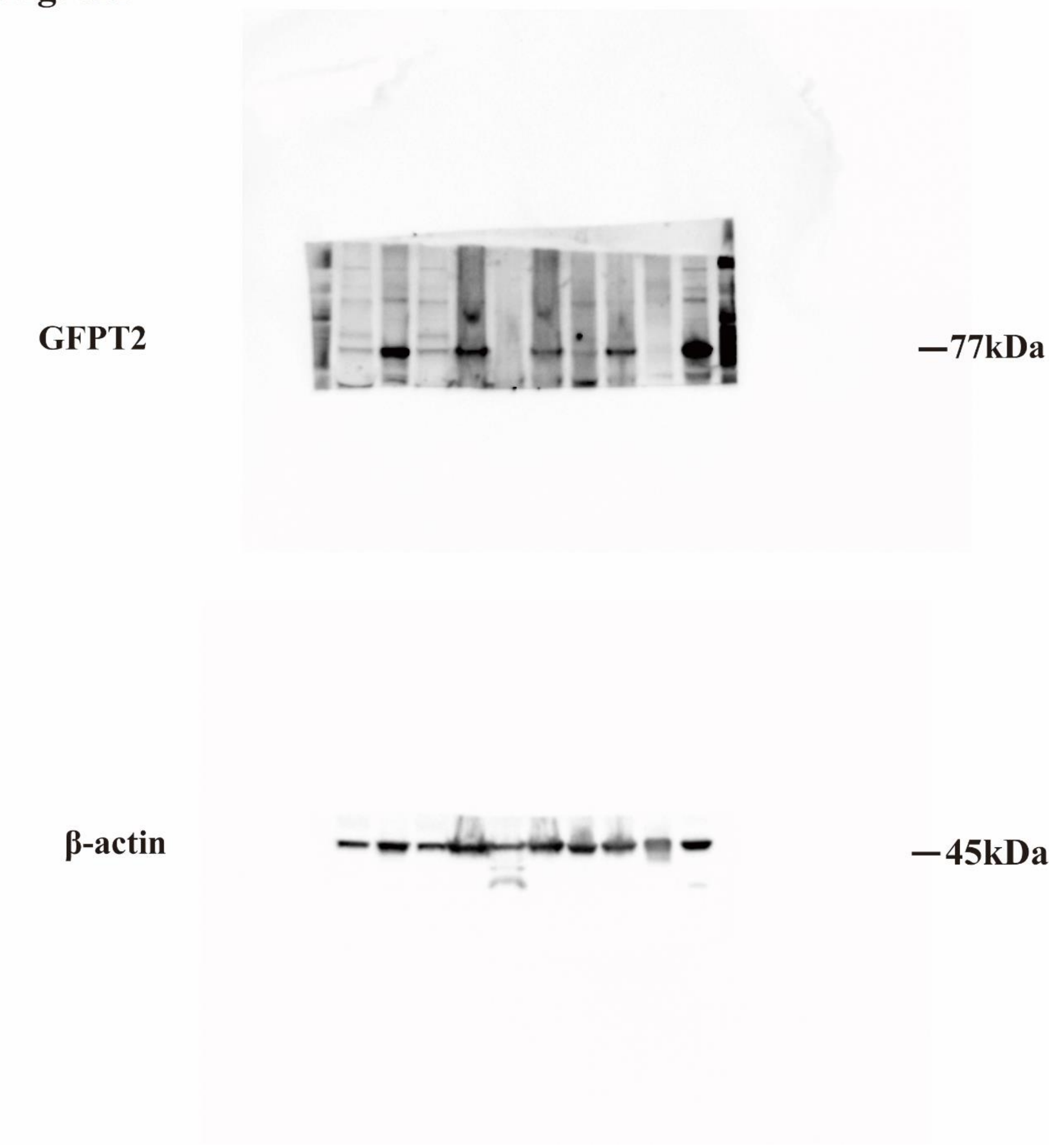

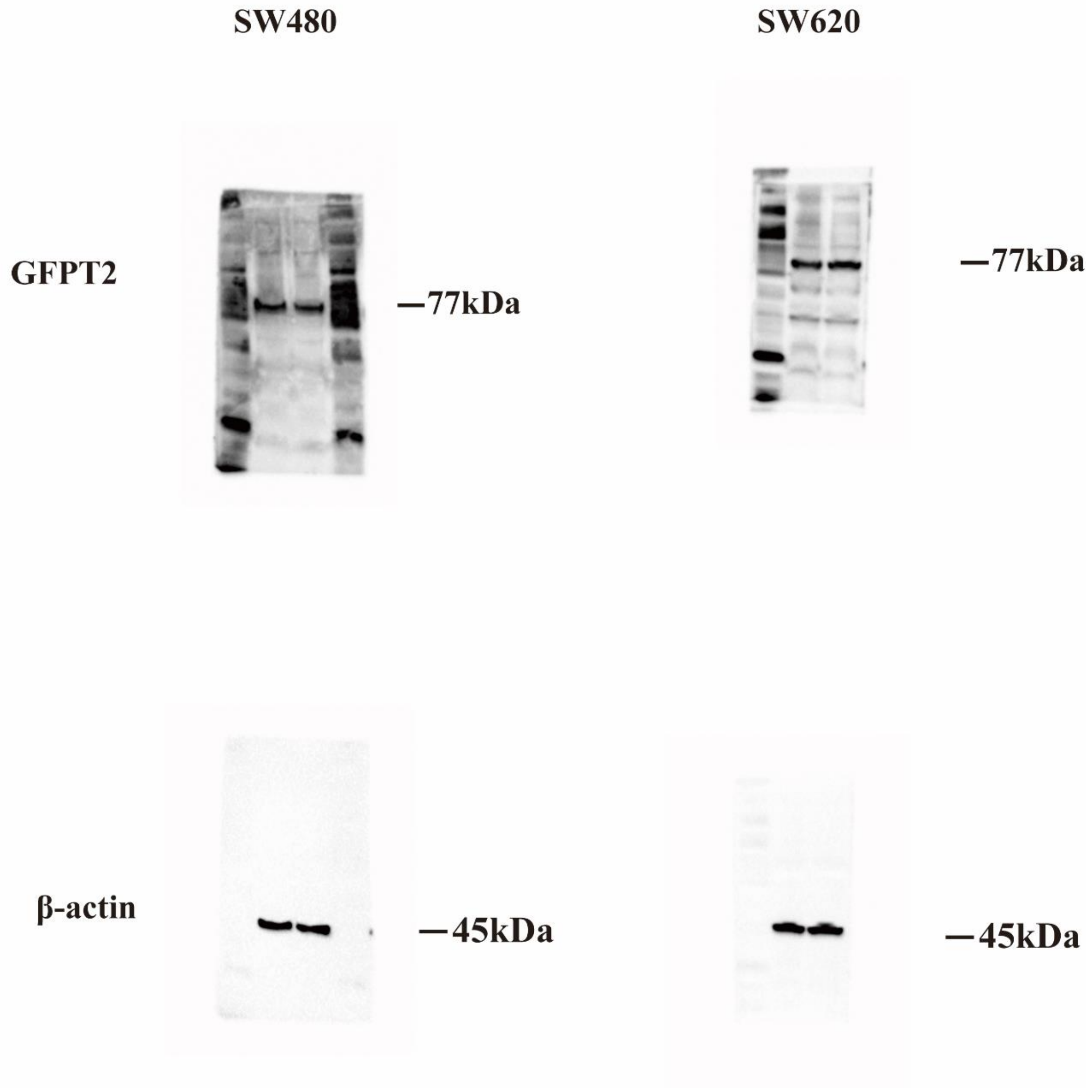

Fig. 4B

Fig. 5E

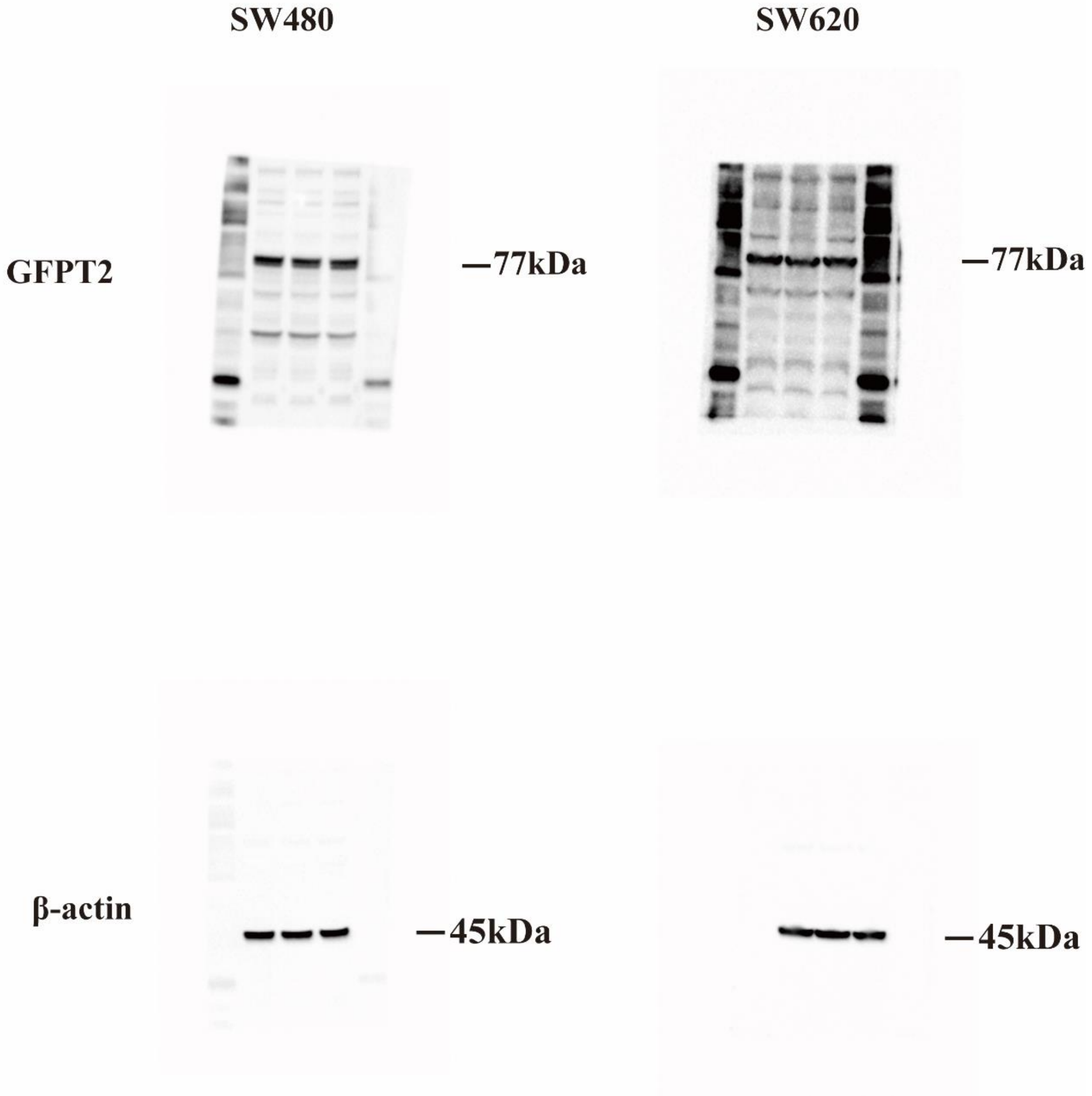

Fig. 6F

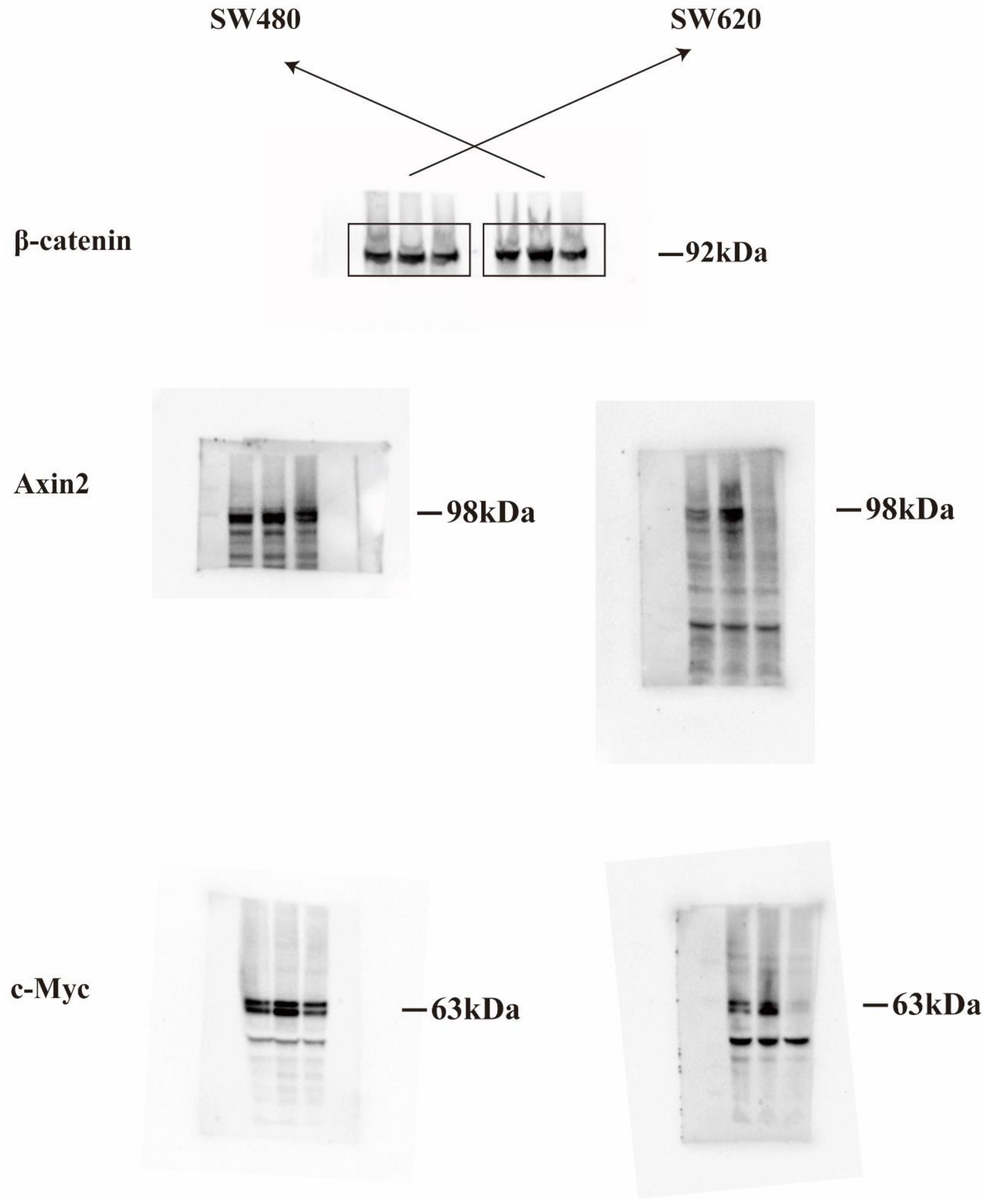

SW480
SW620
β-catenin
92kDa
Axin2
98kDa
98kDa
c-Myc
63kDa
63kDa

Fig. 6F

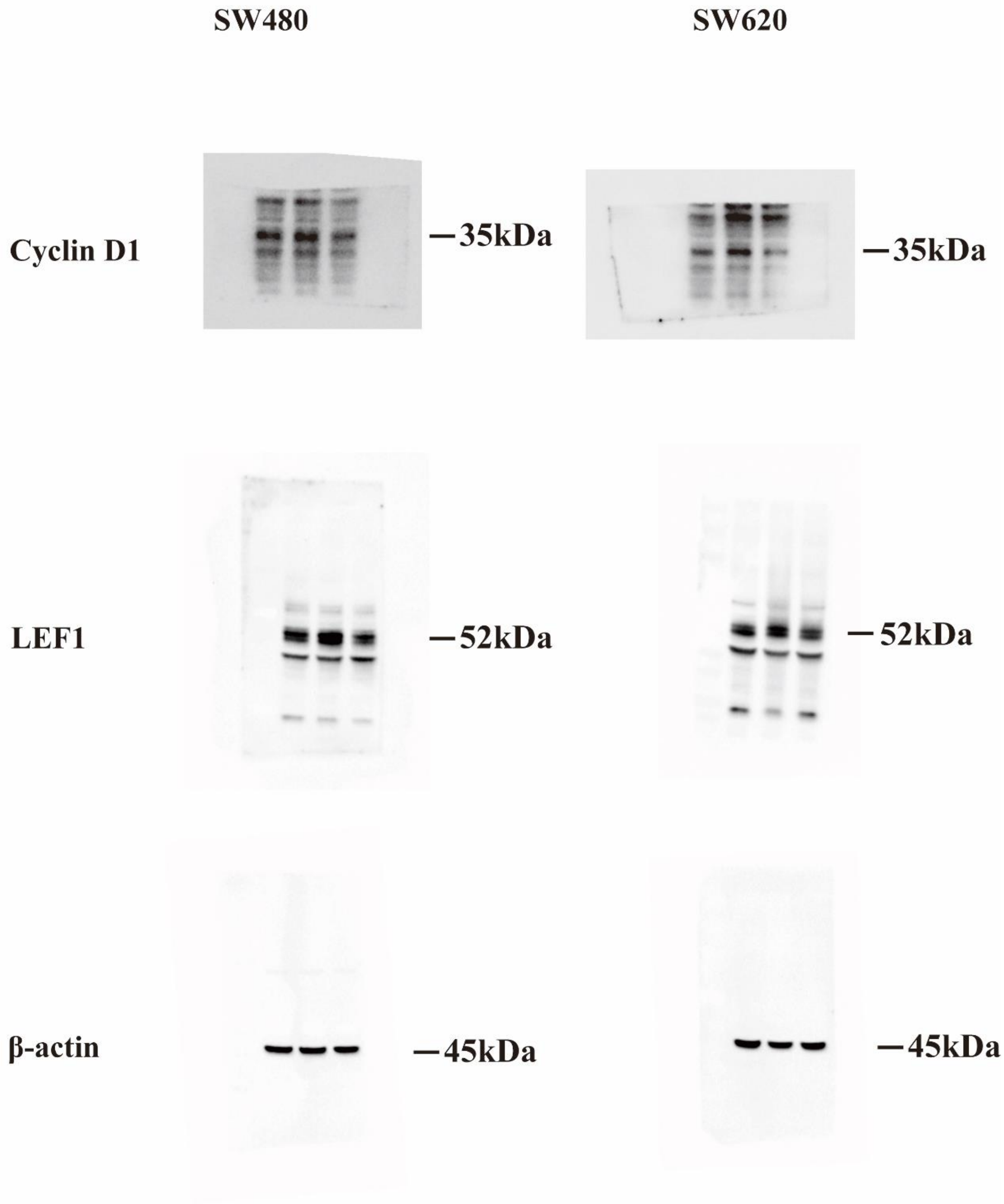

SW480
SW620
Cyclin D1
35kDa
35kDa
LEF1
52kDa
52kDa
β-actin
45kDa
45kDa

Fig. 6G Left panels

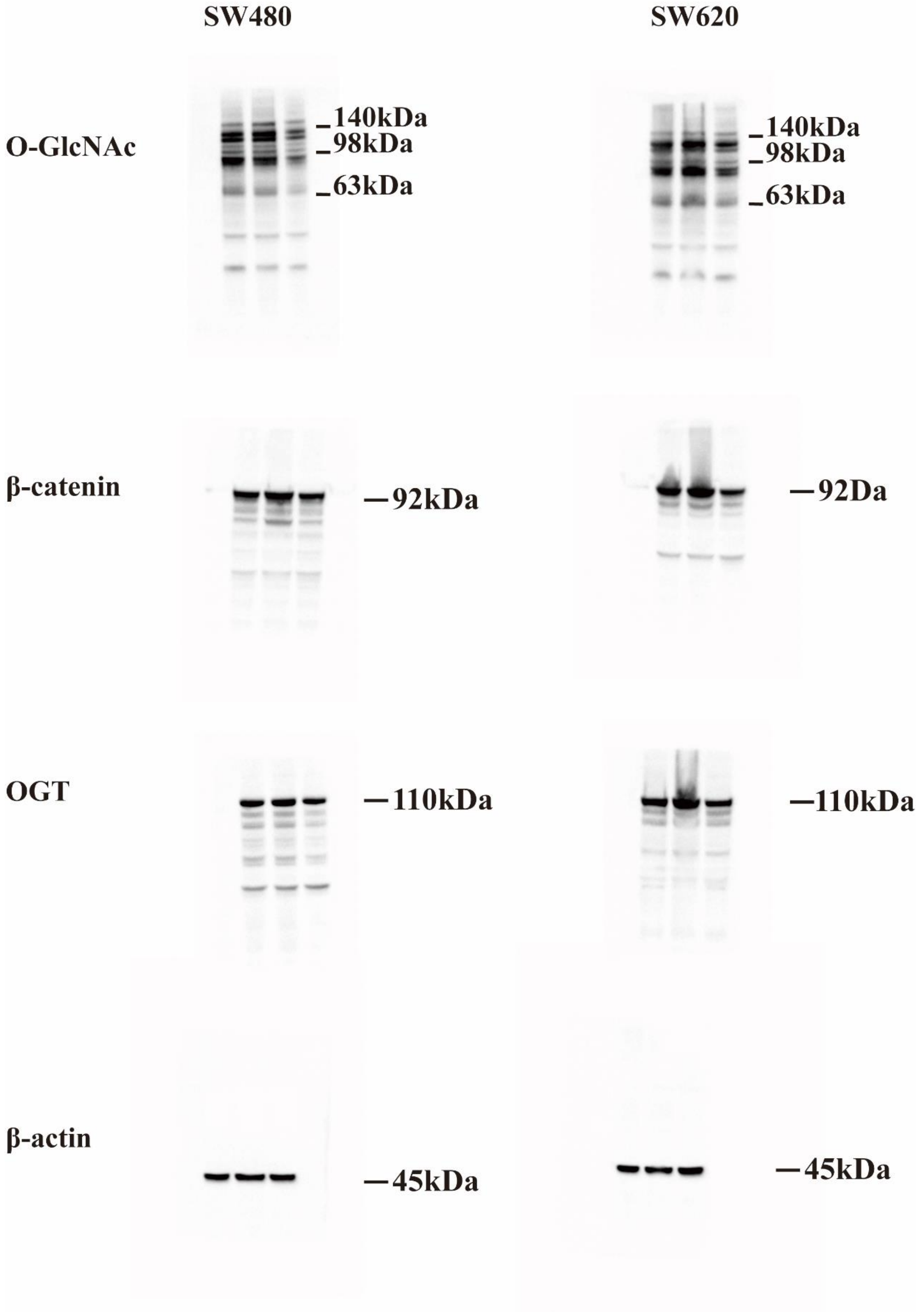

Fig. 6G Right panels

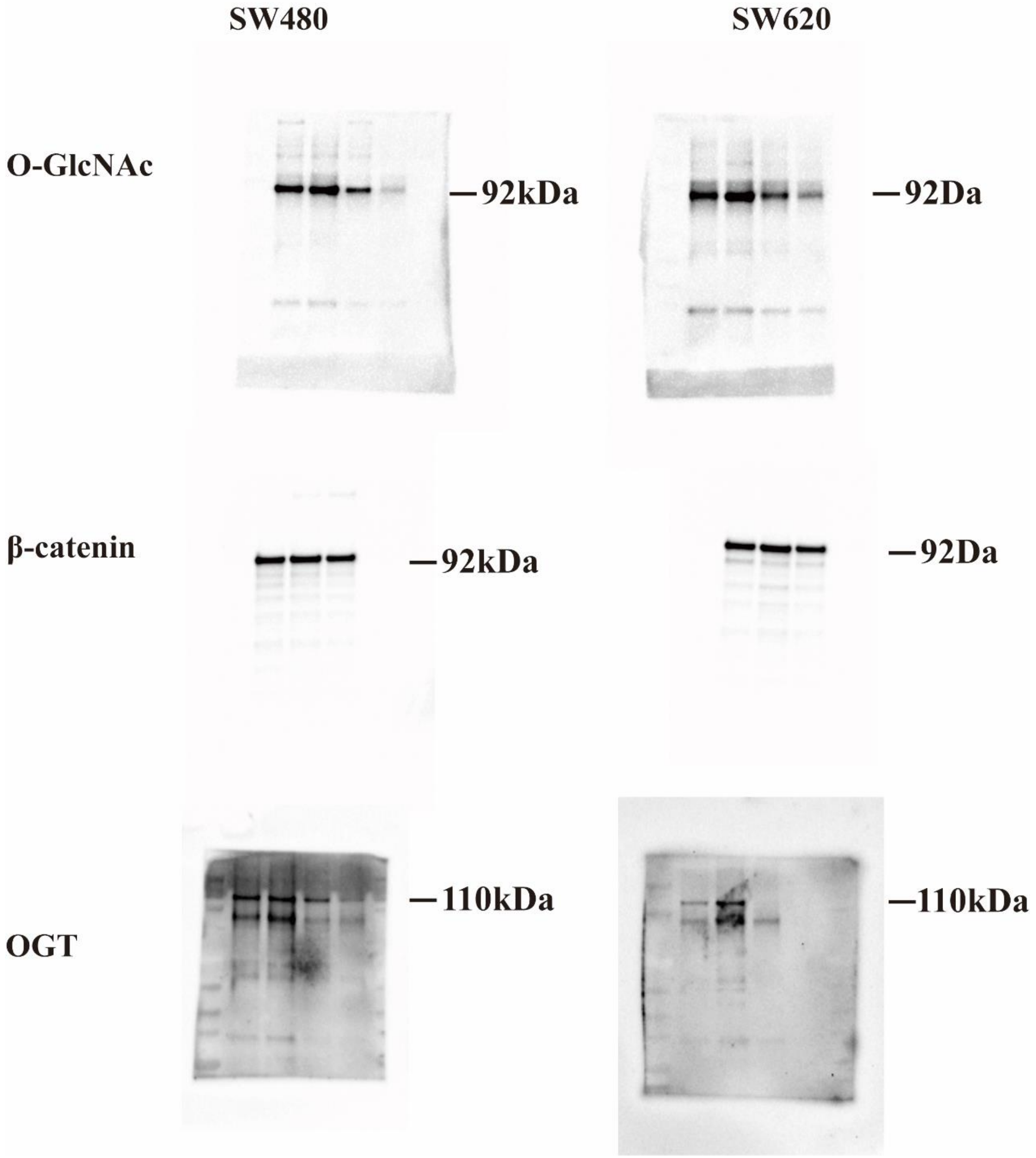

Fig. 7D

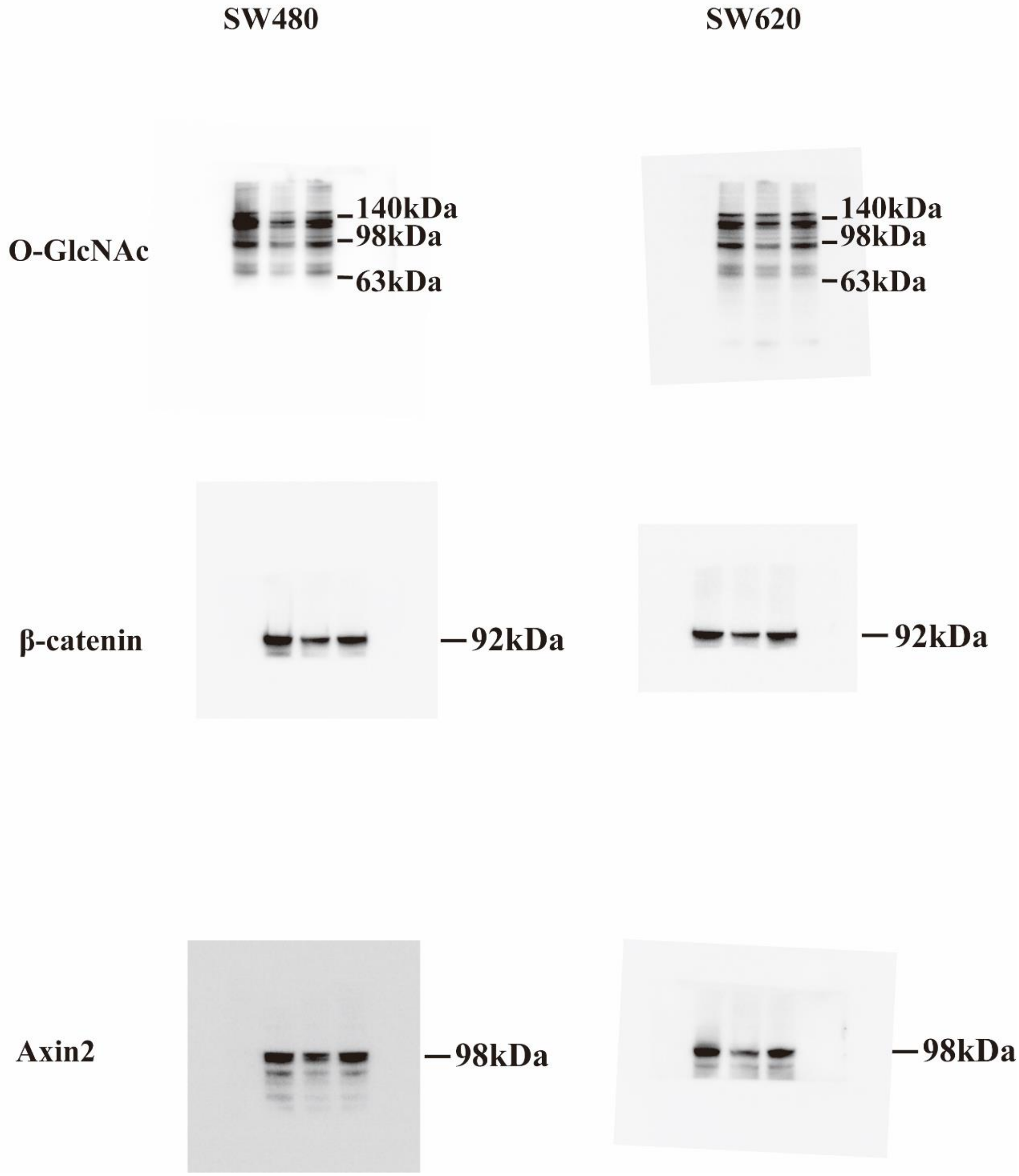
SW480
SW620
O-GlcNAc
140kDa
98kDa
63kDa
140kDa
98kDa
63kDa
β-catenin
92kDa
92kDa
Axin2
98kDa
98kDa

Fig. 7D

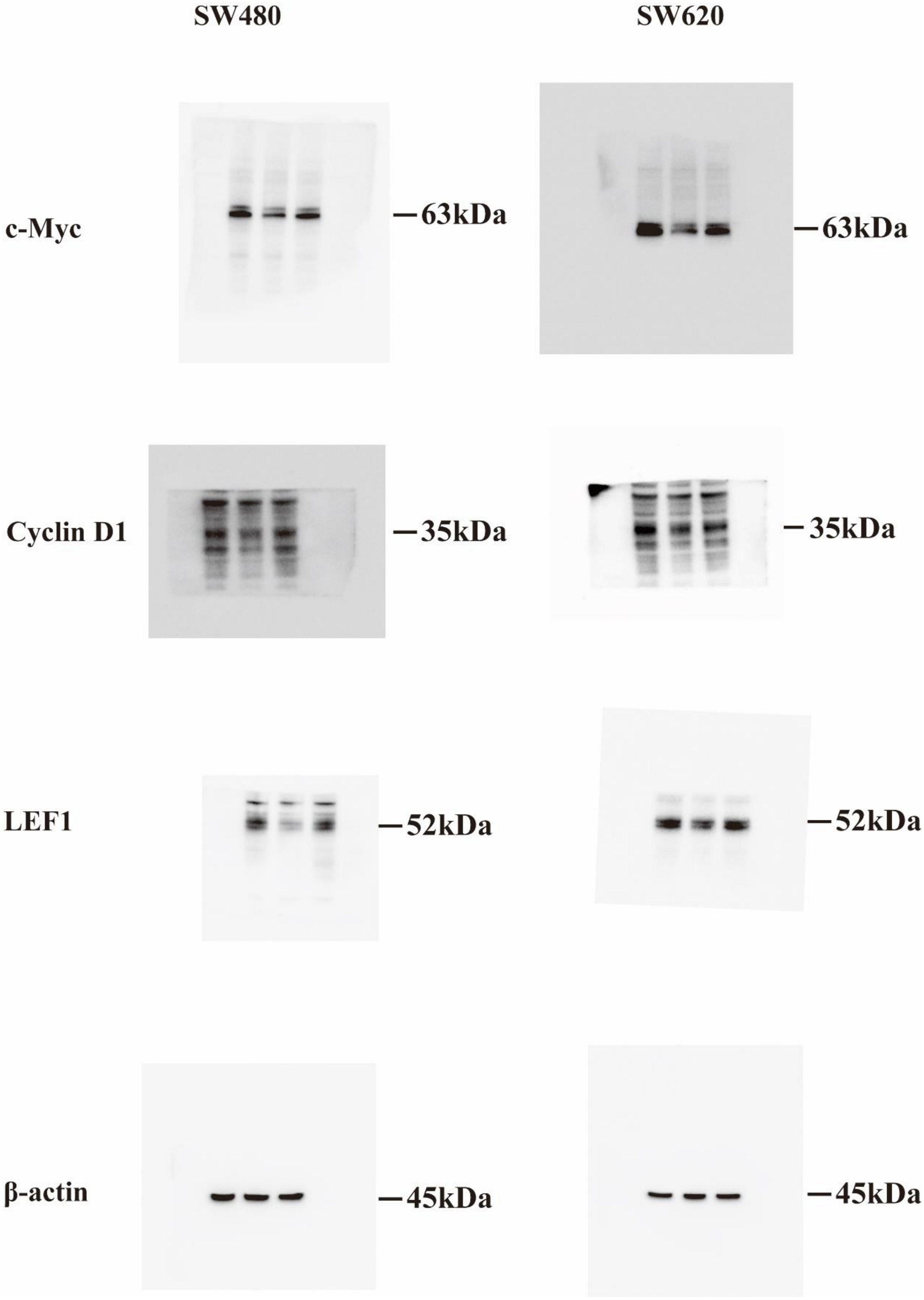
SW480
SW620
c-Myc
63kDa
63kDa
Cyclin D1
35kDa
35kDa
LEF1
52kDa
52kDa
β-actin
45kDa
45kDa

Fig. 8D

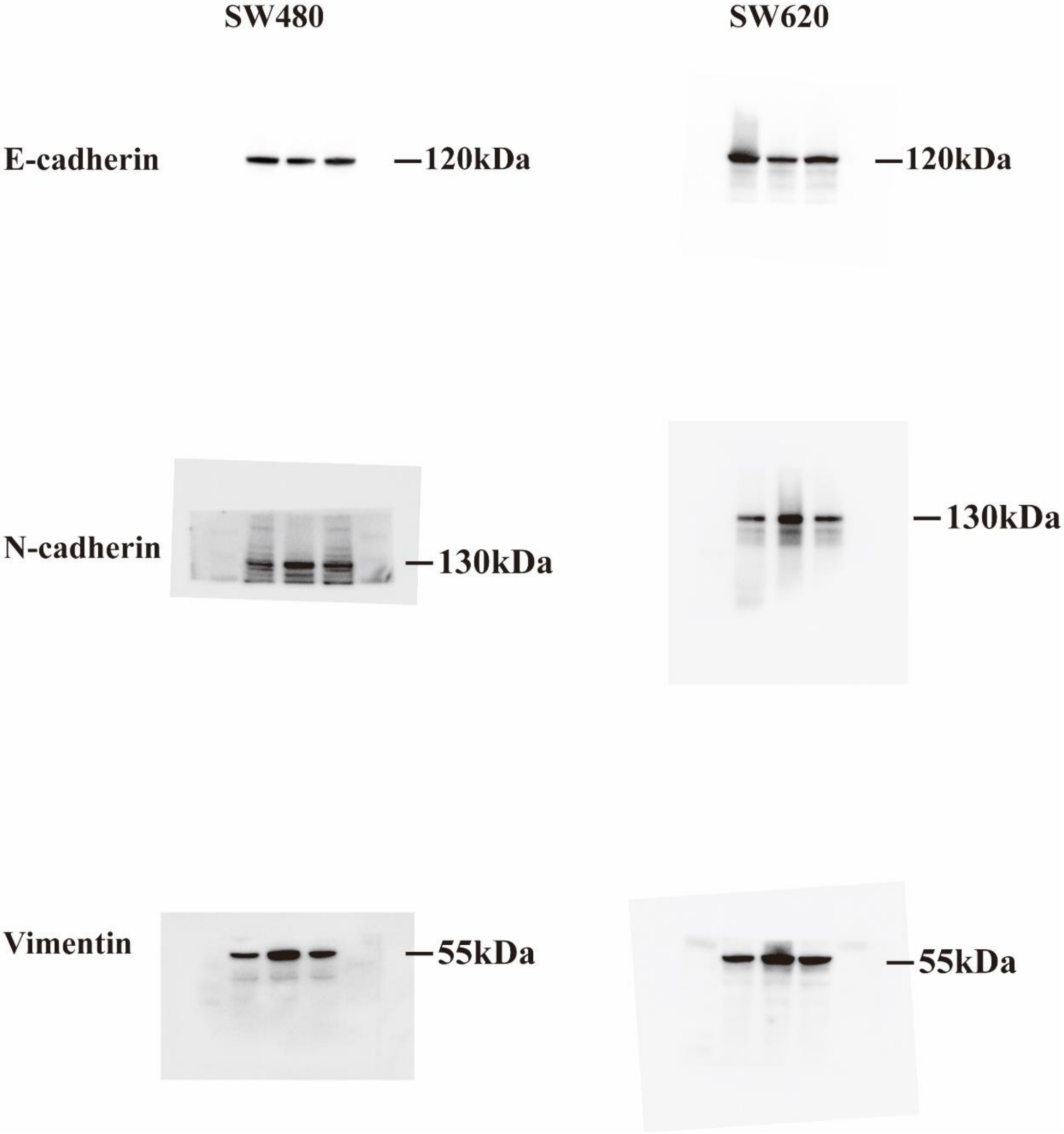
SW480
SW620
E-cadherin
120kDa
120kDa
N-cadherin
130kDa
130kDa
Vimentin
55kDa
55kDa

Fig. 8D

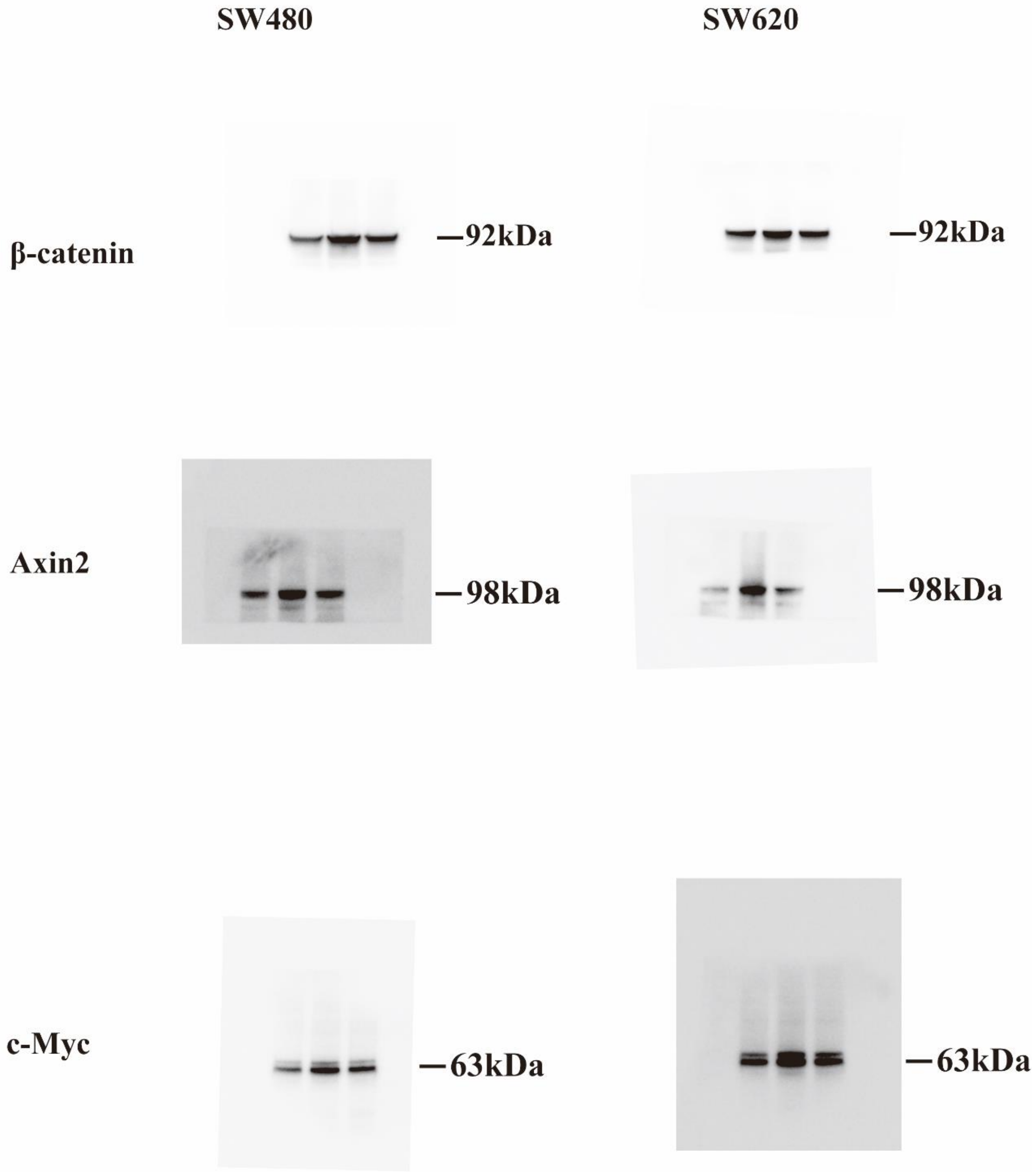
SW480
SW620
β-catenin
92kDa
92kDa
Axin2
98kDa
98kDa
c-Myc
63kDa
63kDa

Fig. 8D

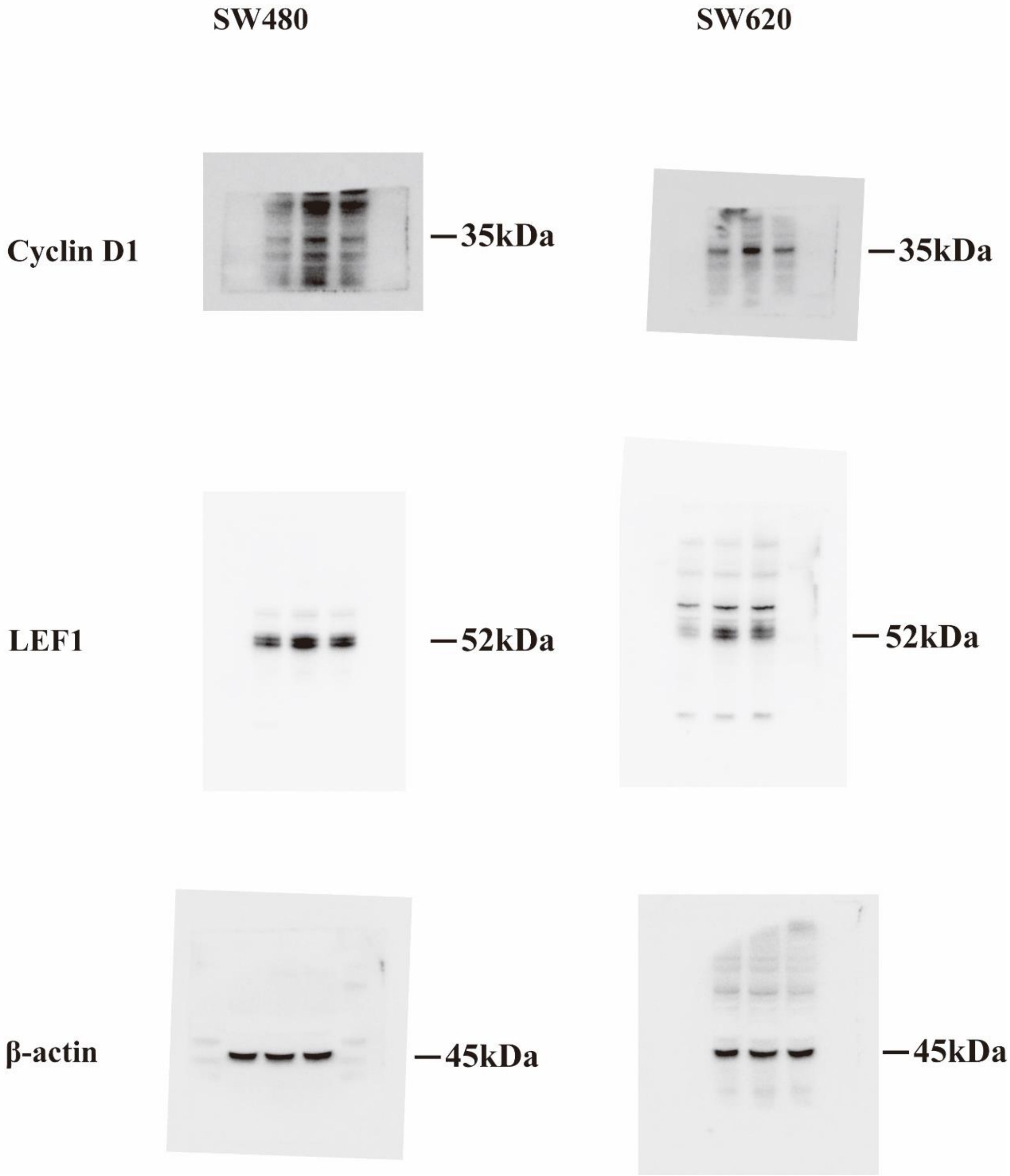
SW480
SW620
Cyclin D1
35kDa
35kDa
LEF1
52kDa
52kDa
β-actin
45kDa
45kDa

Fig. 9F

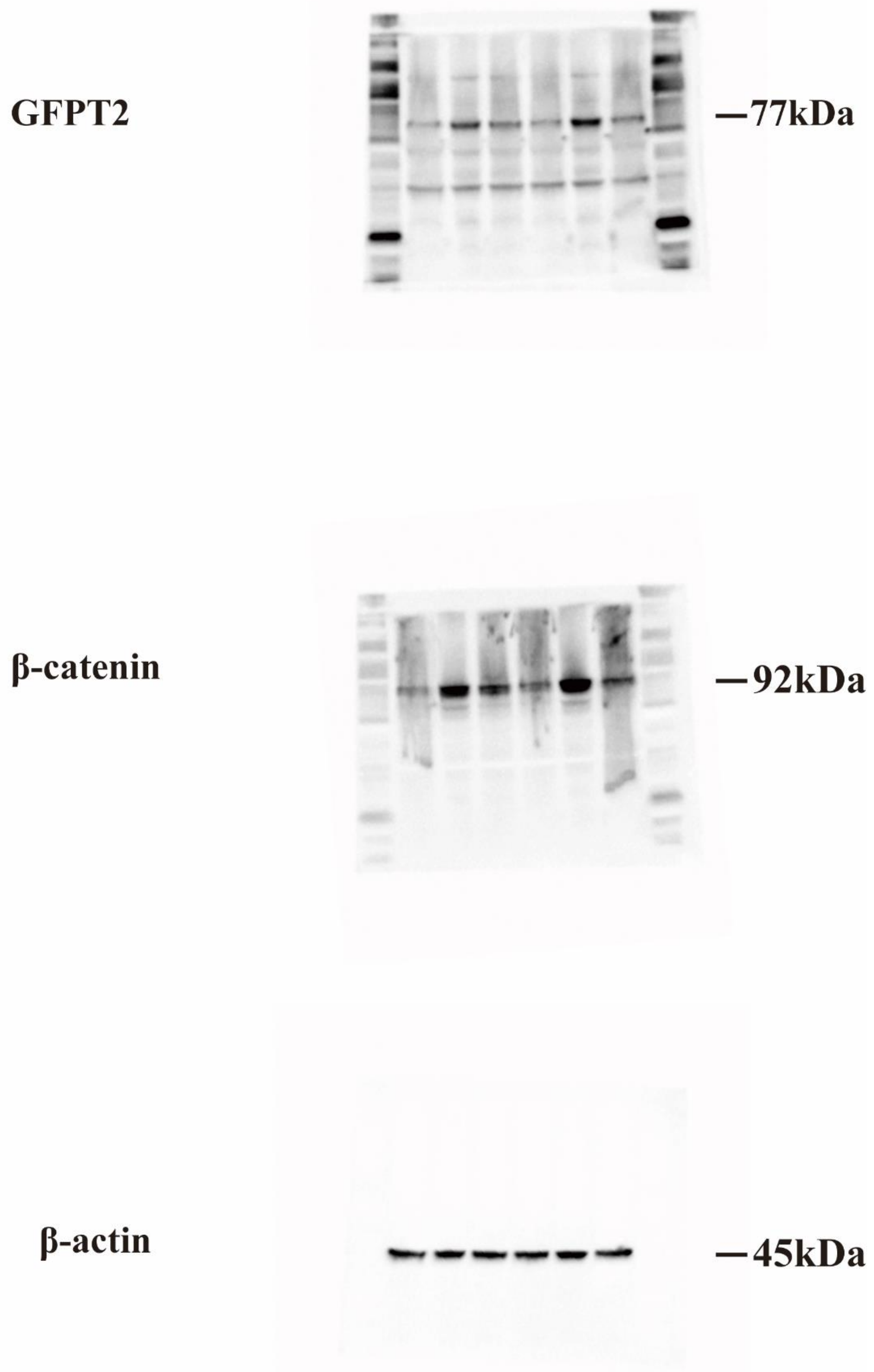

Fig. S2B

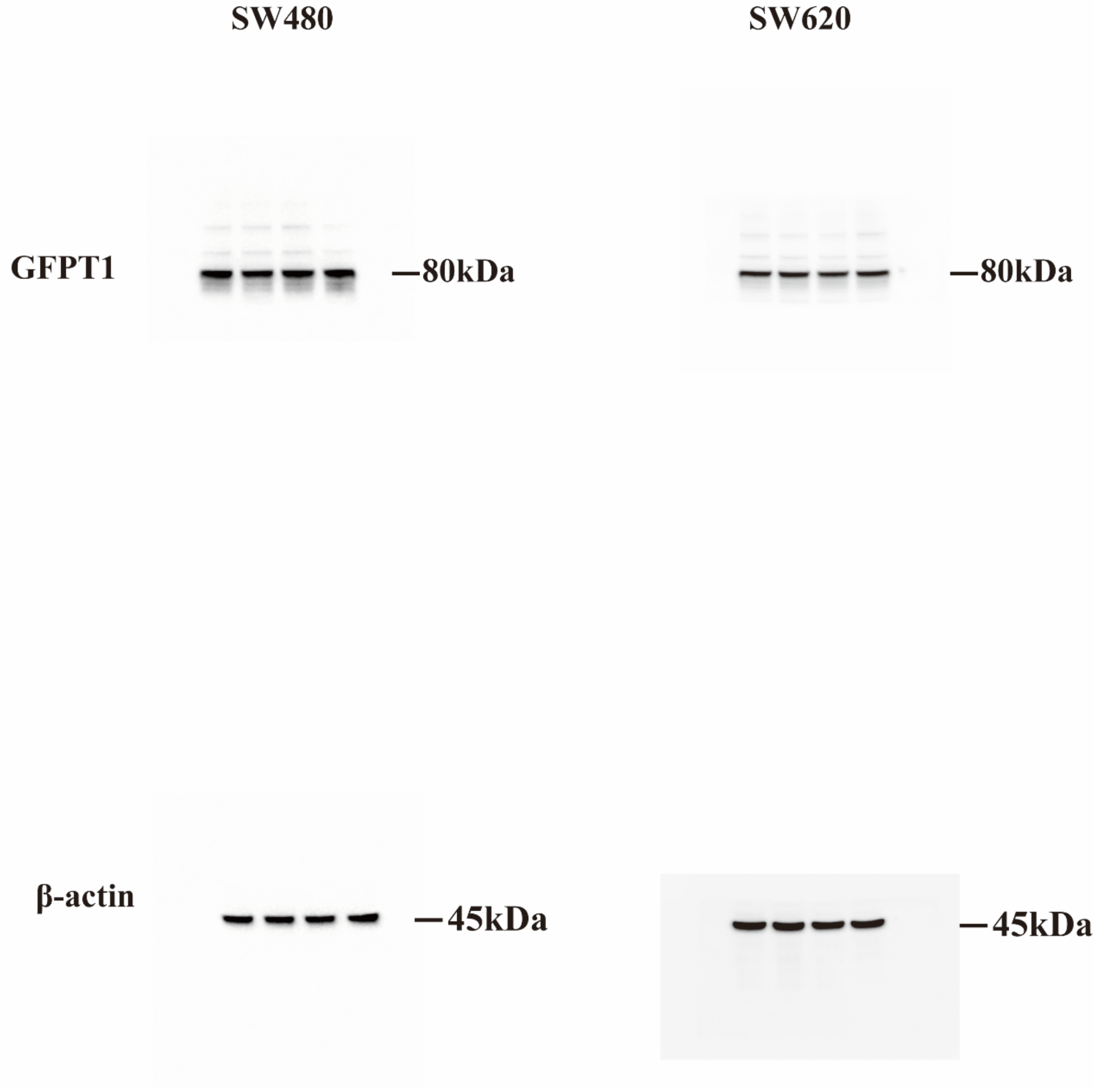

Supplement: Supplementary file 3 — Supplementary Material 3 [file 13062_2026_746_MOESM3_ESM.pdf]
